# Astrocyte‐specific deletion of the mitochondrial *m*‐AAA protease reveals glial contribution to neurodegeneration

**DOI:** 10.1002/glia.23626

**Published:** 2019-04-16

**Authors:** Sara Murru, Simon Hess, Esther Barth, Eva R. Almajan, Désirée Schatton, Steffen Hermans, Susanne Brodesser, Thomas Langer, Peter Kloppenburg, Elena I. Rugarli

**Affiliations:** ^1^ Department of Biology, Institute for Genetics, University of Cologne Cologne Germany; ^2^ Cologne Excellence Cluster on Cellular Stress Responses in Aging‐Associated Diseases (CECAD) University of Cologne Cologne Germany; ^3^ Department of Biology, Institute for Zoology, Biocenter, University of Cologne Cologne Germany; ^4^ Department of Mitochondrial Proteostasis, Max‐Planck‐Institute for Biology of Ageing Cologne Germany

**Keywords:** Bergmann glia, glutamate transporter, mitochondrial disease, necroptosis, neuroinflammation, Purkinje neuron, spinocerebellar ataxia

## Abstract

Mitochondrial dysfunction causes neurodegeneration but whether impairment of mitochondrial homeostasis in astrocytes contributes to this pathological process remains largely unknown. The *m*‐AAA protease exerts quality control and regulatory functions crucial for mitochondrial homeostasis. *AFG3L2*, which encodes one of the subunits of the *m*‐AAA protease, is mutated in spinocerebellar ataxia SCA28 and in infantile syndromes characterized by spastic‐ataxia, epilepsy and premature death. Here, we investigate the role of *Afg3l2* and its redundant homologue *Afg3l1* in the Bergmann glia (BG), radial astrocytes of the cerebellum that have functional connections with Purkinje cells (PC) and regulate glutamate homeostasis. We show that astrocyte‐specific deletion of *Afg3l2* in the mouse leads to late‐onset motor impairment and to degeneration of BG, which display aberrant morphology, altered expression of the glutamate transporter EAAT2, and a reactive inflammatory signature. The neurological and glial phenotypes are drastically exacerbated when astrocytes lack both *Afg31l* and *Afg3l2*, and therefore, are totally depleted of the *m*‐AAA protease. Moreover, mitochondrial stress responses and necroptotic markers are induced in the cerebellum. In both mouse models, targeted BG show a fragmented mitochondrial network and loss of mitochondrial cristae, but no signs of respiratory dysfunction. Importantly, astrocyte‐specific deficiency of *Afg3l1* and *Afg3l2* triggers secondary morphological degeneration and electrophysiological changes in PCs, thus demonstrating a non‐cell‐autonomous role of glia in neurodegeneration. We propose that astrocyte dysfunction amplifies both neuroinflammation and glutamate excitotoxicity in patients carrying mutations in *AFG3L2*, leading to a vicious circle that contributes to neuronal death.

## 1 INTRODUCTION

Astrocytes represent the largest population of glial cells in the mammalian brain. They provide structural and metabolic support for neurons, regulate synapse number and function, maintain the blood–brain barrier, and act as stem cells in the adult brain (Belanger, Allaman, & Magistretti, 2011; Ben Haim & Rowitch, 2017; Chung, Allen, & Eroglu, 2015; Sofroniew & Vinters, 2010). The anatomical and functional relationship between neurons and astrocytes is exemplified by interactions between Purkinje cells (PCs) and Bergmann glia (BG), a specialized form of radial astrocytes in the cerebellum (Bellamy, 2006; De Zeeuw & Hoogland, 2015). BG have an important role in regulating synapse activity and stability, in supporting PCs metabolically, and in uptaking glutamate from the synaptic cleft upon neuronal stimulation. Glutamate uptake by excitatory amino acid transporters (EAAT1 and EAAT2) modulates postsynaptic receptor activation, and protects against extra‐synaptic stimulation and excitotoxicity (Lopez‐Bayghen & Ortega, 2011), which can lead to dark cell degeneration of PCs, a specialized form of cell death characterized by high cytosolic calcium levels and calpain activation (Leist & Jaattela, 2001). This pathogenic mechanism has been proposed to underlie several forms of spinocerebellar ataxia (SCA; Kasumu & Bezprozvanny, 2012), which are neurological diseases characterized by loss of balance, progressive ataxia and dysarthria, and caused by degeneration of the cerebellum and its afferent and efferent connections (Taroni & DiDonato, 2004).

*AFG3L2* is mutated in a dominant form of SCA28 (Cagnoli et al., 2006, 2010; Di Bella et al., 2010; Smets et al., 2014), and in early‐onset recessive syndromes characterized by severe spastic‐ataxia, epilepsy, and premature death (Eskandrani et al., 2017; Pierson et al., 2011). AFG3L2 is abundantly expressed in the brain and can homo‐oligomerize to form a functional proteolytic complex, the *m‐*AAA protease, which is embedded in the inner mitochondrial membrane. In addition, AFG3L2 can assemble in functional hetero‐oligomeric complexes with the homologous subunit SPG7 (also called paraplegin; Banfi et al., 1999; Casari et al., 1998; Koppen, Metodiev, Casari, Rugarli, & Langer, 2007). The *SPG7* gene is in turn responsible for an autosomal recessive form of hereditary spastic paraplegia, often associated with ataxia (Casari et al., 1998). The *m‐*AAA protease is a core component of a quality control system in mitochondria, which degrades misfolded polypeptides and proteins lacking assembly partners on the matrix side of the inner mitochondrial membrane. Moreover, this complex performs essential regulatory roles by exerting proteolytic maturation of specific mitochondrial proteins (Patron, Sprenger, & Langer, 2018; Rugarli & Langer, 2012).

The importance of an intact *m‐*AAA protease for neuronal function and survival has been highlighted by studies in mouse models. Full‐body *Afg3l2* knock‐out mice manifest a severe neurological phenotype, characterized by axonal developmental failure, and die within the first 2 weeks of age (Maltecca et al., 2008). Post‐natal PC‐specific deletion of *Afg3l2* triggers mitochondrial fragmentation and neuronal death (Almajan et al., 2012). In contrast, mice carrying heterozygous *Afg3l2* mutations are viable and display a late‐onset, mild ataxic phenotype (Maltecca et al., 2009), probably because of the presence in the mouse of a redundant homologous gene, *Afg3l1* (Koppen et al., 2007; Kremmidiotis et al., 2001). PCs in these mouse models show features of dark cell degeneration, a phenotype reminiscent of that induced by glutamate excitotoxicity (Maltecca et al., 2009). Remarkably, the ataxia in *Afg3l2* heterozygous mice is ameliorated by administration of the antibiotic ceftriaxone, which promotes synaptic glutamate clearance by increasing the expression of the glutamate receptor EAAT2 in astrocytes (Maltecca et al., 2015). This finding suggests that astrocytes surrounding PCs may play a yet unrecognized role in the pathogenesis of SCA28 and other *AFG3L2*‐linked neurological syndromes.

Here, we have inactivated *Afg3l2* alone or together with the homologue *Afg3l1* in murine astrocytes to answer two fundamental questions: (a) Do astrocytes require a functional *m‐*AAA protease in vivo? (b) Does astrocyte‐specific *m*‐AAA protease deficiency impair neuronal function and contribute to neurodegeneration in a non‐cell‐autonomous way? Surprisingly, our data demonstrate that BG functionally depend on the *m‐*AAA protease. Moreover, deficiency of the *m*‐AAA protease in astrocytes changes morphological and electrophysiological properties of PCs. We propose a pathogenic model for SCA28 where neurons are the primary hit of the pathogenic process, while astrocyte dysfunction amplifies and precipitates neuronal damage.

## 2 METHODS

### 2.1 Mouse models

All animal experiments were performed in accordance with European (EU directive 86/609/EEC), German (TierSchG) and institutional guidelines. They were also approved by the local authorities (Landesamt für Natur, Umwelt und Verbraucherschutz Nordrhein‐Westfalen, Germany). The astrocyte‐specific *Afg3l2* knock‐out mouse line (astro‐L2 KO) was generated by crossing the conditional *Afg3l2* ^*fl/fl*^ mice (Almajan et al., 2012) with the inducible GFAP‐Cre‐ER™ mice (GFAP‐Cre^tg/wt^; Chow, Zhang, & Baker, 2008). The controls used were *Cre* negative (GFAP‐Cre^wt/wt^, Ctrl) littermates. Unless specified, both male and female mice were used for experiments. To visualize mitochondria in astrocytes, animals carrying the GFAP‐Cre^tg/wt^ transgene were crossed with the ROSA26^+/SmY^ mice (mtYFP^tg/+^; Sterky, Lee, Wibom, Olson, & Larsson, 2011). To induce *Cre*‐mediated recombination, lactating mothers received a daily intraperitoneal injection of tamoxifen (1 mg/10 g body‐weight) for five consecutive days, starting at postnatal Day 7. Tamoxifen (T5648, Sigma) was dissolved in a mixture 9:1 of corn oil and pure ethanol, to the final concentration of 20 mg/ml. To generate a mouse model with total ablation of the *m*‐AAA protease (astro‐DKO), we crossed the *Afg3l2* ^fl/fl^: GFAP‐Cre^tg/wt^ mice with the *Afg3l1* full body knock‐out mice (*Afg3l1* ^−/−^; Wang et al., 2016). The recombination was induced as described above. Both males and females were used for the experiments. *Afg3l1* ^−/−^ (L1^−/−^) littermates were used as controls. Mice were born and maintained in ventilated cages, under a 12 hr dark/light cycle and regularly monitored for signs of distress or pain. A regular chow diet (Ssniff V1554‐330) was provided ad libitum.

### 2.2 Behavioral studies

Motor coordination was tested using a Rotarod apparatus (TSE system). Briefly, male and female mice were trained for 1 min on the accelerating rod. Then, the latency to fall from the rotating rod was measured for a maximum of 300 s. Each testing day consisted of three trials, performed 15 min apart. The ataxic score evaluation was performed as previously described (Guyenet et al., 2010). Briefly, the assessment consisted of four tests: ledge test, hindlimb clasping, gait, and kyphosis evaluation. Each test was scored between 0 and 3 depending on the severity of the phenotype. The score was assigned by summing up the single‐test scores.

### 2.3 Immunofluorescence and quantification

Mice were deeply anesthetized via intraperitoneal injection of xylazine/ketamine (10 mg/100 mg per kg of body‐weight) and transcardially perfused with 4% paraformaldehyde (PFA) in phosphate‐buffered saline. The brain was then removed and postfixed in 4% PFA for 24 hr. The cerebellum was dissected, embedded in 6% agar and cut in 30 μm sagittal sections using a vibratome (VS1000, Leica). Free‐floating sections were permeabilized and blocked in 0.4% Triton X‐100 and 10% goat serum in Tris‐buffered saline (TBS) at room temperature (RT) for 1 hr. Primary antibody incubations were performed overnight (O/N) at 4°C, while secondary antibody incubations were performed at RT for 2 hr. Sections were mounted on slides using FluorSave reagent (Calbiochem). Fluorescence images were acquired using an Axio‐Imager M2 microscope equipped with Apotome 2 (Zeiss) and processed using the software AxioVision SE64 Rel. 4.9.1. The following antibodies were used for immunofluorescence: S100 (DAKO, Z0311; 1:2,000); SOX2 (Abcam, 979559; 1:700); GFP (Aves, GFP‐1020; 1:500); GFAP (Cell Signaling, 3670; 1:500); Iba1 (Wako, 019‐19741; 1:2,000); EAAT1 (Miltenyi, 5684; 1:1,000); EAAT2 (BD Transduction, 611654; 1:1,000); Calbindin (SWANT, 300; 1:500); SMI31 (Covenance, SMI‐31R; 1:500). As secondary antibodies, we used: anti‐mouse Alexa Fluor 488 (A‐11029), Alexa Fluor 546 (A‐21143), and Alexa Fluor 594 (A‐11005); anti‐rabbit Alexa Fluor 488 (A‐11034), Alexa Fluor 546 (A‐11035), and Alexa Fluor 594 (A‐21207) from Molecular Probes; anti‐chicken Alexa Fluor 488 (703‐545‐155, Jackson ImmunoResearch). S100^+^ cells located in the PC layer (PCL) and the molecular layer (ML) were manually counted using ImageJ. For each mouse, results obtained from 6 to 7 images per cerebellum were averaged and normalized to the area. The *n* is indicated in the respective figure. For these experiments, some mice analyzed carried also the mtYFP allele.

### 2.4 Electron microscopy

Cerebella from perfused animals were postfixed in 2% glutaraldehyde (Sigma), followed by treatment with 1% osmium tetroxide (Sigma) and dehydration in ethanol and propylene oxide. Samples were then embedded in Epon (Fluka) and 70 nm sections were cut using an ultramicrotome (EM UC7, Leica). The sections were stained with uranyl acetate (PlanoGMBH) and lead citrate (Electron Microscopy Sciences). Images were taken by a transmission electron microscope (JEOL JEM2100PLUS) equipped with GATAN OneView camera.

### 2.5 Immunohistochemistry and quantification

Animals were sacrificed via cervical dislocation. The cerebella were immediately immersed in 4% PFA for 24 hr and embedded in paraffin. Five micrometer sagittal sections were cut using a microtome (RM2255, Leica). Following deparaffinization, epitope retrieval was performed by boiling the sections in 0.1M citrate buffer (pH 6). The sections were then blocked and permeabilized in 1% albumin, 0.1% Triton X‐100, and 0.05% Tween‐20. Sections were incubated with primary antibody against cleaved caspase 3 Asp175 (Cell Signaling, 9661; 1:500) O/N at 4°C, and with secondary biotinylated anti‐rabbit antibodies (Vector, BA‐1000). The staining was visualized using the ABC Kit Vectastain Elite and DAB substrate (Vector Laboratories). Three animals at each time‐point were used for each genotype. Positively stained cells were manually counted from 3 to 4 cerebellar sections/animal.

### 2.6 RNA extraction and quantitative real time‐polymerase chain reaction

Cerebella were lysed and total RNA was extracted using TRIzol reagent. SuperScript First‐Strand Synthesis system (Invitrogen) was used to retro‐transcribe 2 μg of RNA, according to the manufacturer′s protocol. Quantitative polymerase chain reaction was performed with SYBR green Master mix (Thermo Fisher Scientific) and the fold increase was determined with the formula 2^(−ΔΔCt)^. *Hprt1* was used for normalization. Primer sequences are listed in the Supporting Information.

### 2.7 Amino acid quantification

Cerebella were snap frozen after cervical dislocation. Endogenous amino acid levels were determined by Liquid Chromatography coupled to Electrospray Ionization Tandem Mass Spectrometry (LC‐ESI‐MS/MS). The detailed experimental procedure is provided in the Supporting Information.

### 2.8 Western blot

Cerebella were lysed in RIPA buffer (50 mM Tris–HCl pH 7.4, 150 mM NaCl, 5 mM EDTA, 1% Triton X‐100, 1% sodium deoxycholate, 0.1% SDS) supplemented with protease cocktail inhibitor (Sigma). Fifty micrograms of proteins were loaded on a 10% sodium dodecyl sulfate–polyacrylamide gel electrophoresis. Proteins were then transferred to Polyvinylidene fluoride membranes (GE Healthcare, Life science). The following primary antibodies were diluted in 5% milk in TBS containing 0.1% Tween‐20: RIPK3 (Cell Signaling, 15828S; 1:1,000), Pan‐Actin (Millipore, MAB1501R, 1:4,000).

### 2.9 Electrophysiology

Perforated patch‐clamp experiments were performed on sagittal cerebellar slices (300 μm) from male and female astro‐DKO mice and L1^−/−^ littermates (25–33 days of age). Experiments were carried out essentially as described previously (Almajan et al., 2012). Recorded neurons were labeled with biocytin‐streptavidin. The detailed procedure is described in Supporting Information.

### 2.10 Morphological analysis of PCs

For morphological analysis of PCs, the “Simple Neurite Tracer” plug‐in of ImageJ Ver. 2.0.0‐rc‐69/1.52i was used.

### 2.11 Statistical analysis

All graphs display the mean ± standard deviation (*SD*) or the standard error of the mean (*SEM*), as indicated. If not stated otherwise statistical comparison of the means of two groups was performed using a two‐tailed unpaired Student's *t* test. A *p* value of less than .05 was considered significant. Statistical analysis was performed using GraphPad Prism software, 6.07 and 8. The single cell electrophysiological and morphological data were tested for outliers via the ROUT function (*Q* = 1%) of GraphPad Prism 8.

## 3 RESULTS

### 3.1 Astrocyte‐specific deletion of *m*‐AAA protease subunits causes neurological phenotypes

**Figure 1:**
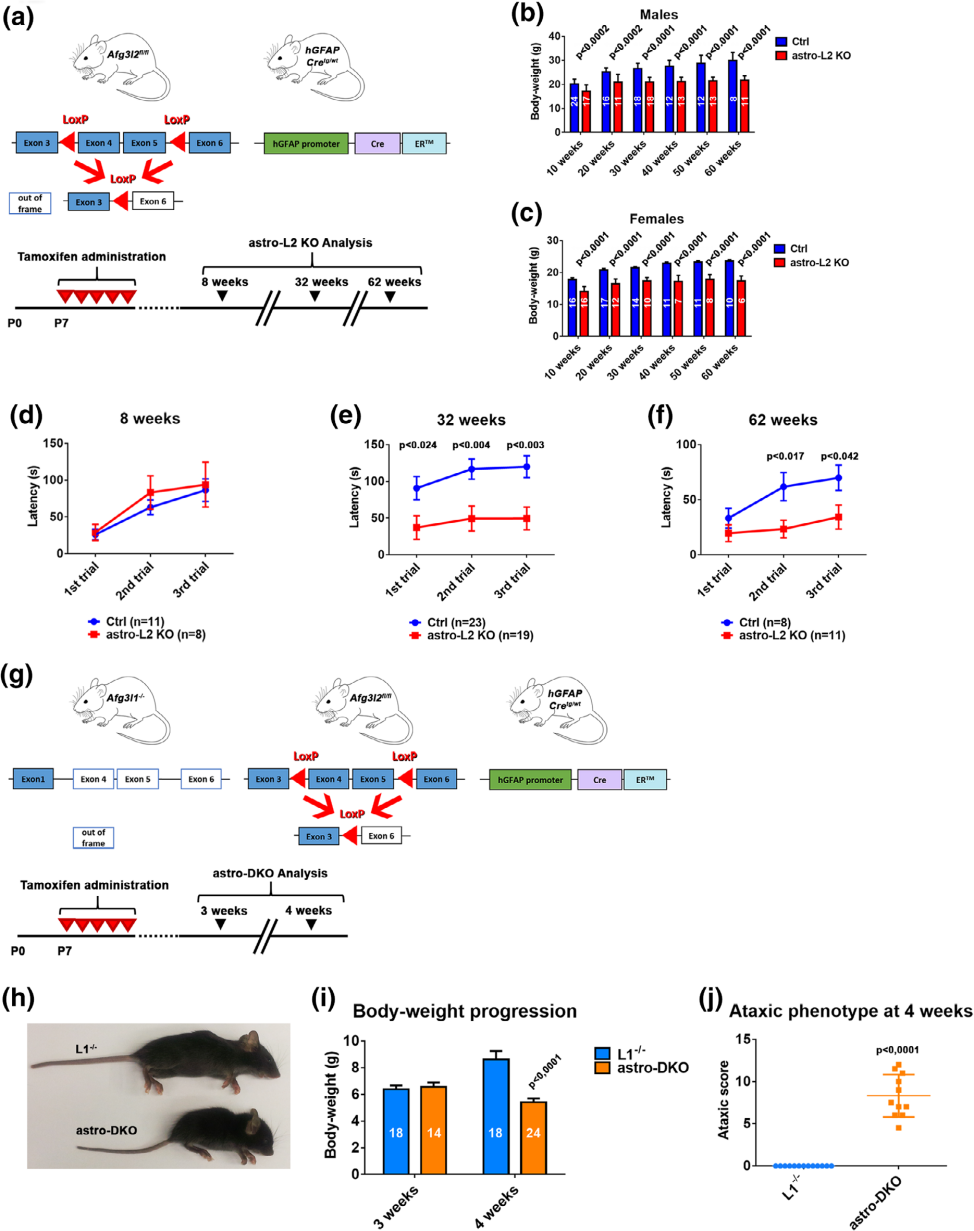
Astrocyte‐specific mouse models with deficiency of the *m*‐AAA protease show a neurological phenotype. (a) Experimental set up to obtain astro‐L2 KO mice. Lactating mothers (*Afg3l2* ^*fl/fl*^) received intraperitoneal injections of tamoxifen as outlined in the scheme. Analyses on the litters were performed at 8, 32, or 62 weeks. (b, c) Body‐weight comparison of male and female astro‐L2 KOs and Ctrls. *n* is indicated in each column. (d–f) Time spent on the rotarod apparatus before falling at different time‐points. (g) Experimental set up to obtain astro‐DKO mice. Lactating mothers (*Afg3l1* ^−/−^:*Afg3l2* ^*fl/fl*^) received intraperitoneal injections of tamoxifen as outlined in the scheme. Analyses on the litters were performed at 3 and 4 weeks. (h) Picture representing a L1^−/−^ and an astro‐DKO animal at 4 weeks of age. (i) Body‐weight comparison of L1^−/−^ and astro‐DKO mice at 3 and 4 weeks. (j) Ataxic score of L1^−/−^ and astro‐DKOs at 4 weeks. In all graphs, data represent means ± *SEM. p* values were obtained by Student's *t* test [Color figure can be viewed at wileyonlinelibrary.com]

To explore the function of the *m‐*AAA protease in astrocytes, we developed two novel mouse models in which the *Afg3l2* gene, alone or together with the redundant *Afg3l1* paralogue, was specifically deleted in astrocytes postnatally. *Afg3l1* is highly homologous to *Afg3l2* in the mouse genome and has similar functional properties but a different expression pattern (Koppen et al., 2007; Martinelli et al., 2009), while it is a pseudogene in humans (Kremmidiotis et al., 2001). *Afg3l1* knock‐out mice have no detectable phenotype up to 1 year of age (Wang et al., 2016). However, the expression of *Afg3l1* in the mouse mitigates phenotypes associated to the lack of *Afg3l2* in oligodendrocytes (Wang et al., 2016). Furthermore, *Afg3l1* and *Afg3l2* are expressed in astrocytes at comparable levels as in neurons (Wang et al., 2016; Zhang et al., 2014). To produce the first model, we crossed mice carrying *Afg3l2* alleles with LoxP flanked exons 4 and 5 (*Afg3l2* ^*fl/fl*^; Almajan et al., 2012) with a transgenic line that expresses a tamoxifen‐inducible form of the *Cre* recombinase under the human glial fibrillary acidic protein (GFAP) promoter (GFAP‐Cre‐ER™ line; Chow et al., 2008; Figure 1a). To avoid targeting neural precursors, we administered tamoxifen at postnatal day 7 (P7) by injecting lactating mothers for five consecutive days (Figure 1a; Silbereis, Cheng, Ganat, Ment, & Vaccarino, 2009). This treatment results in efficient and selective targeting of BG in the cerebellum of the pups, as we established by the mean of a transgenic reporter line that expresses a mitochondrially targeted fluorescent protein (mtYFP) upon *Cre* recombination (Sterky et al., 2011; Figure S1A,B). As there is no available antibody against AFG3L2 that works for immunofluorescence, we confirmed deletion of exons 4 and 5 of *Afg3l2* by amplification of the surrounding region from genomic DNA isolated from the cerebellum and forebrain (Figure S1C).

Astrocyte‐specific *Afg3l2* knock‐out (from now on labeled as astro‐L2 KO, genotype: *Afg3l2* ^*fl/fl*^: GFAP‐Cre^tg/wt^) of both sexes were born at expected Mendelian ratio and displayed reduced weight in respect to control littermates (Ctrl, genotype: *Afg3l2* ^*fl/fl*^) at all ages analyzed (Figure 1b,c). To unravel a possible neurological phenotype, we tested the motor abilities of the mice on an accelerating rotarod apparatus in young (8 weeks), adult (32 weeks), and old (62 weeks) mice. Astro‐L2 KO males performed similarly to Ctrl littermates at 8 weeks, but spent significant less time on the rotating rod at 32 and 62 weeks of ages (Figure 1d–f). In contrast, the rotarod performance of astro‐L2 KO female mice did not show a consistent difference compared to Ctrl littermates (Figure S2). As there are previous reports in the literature that indicate sex‐specific differences in phenotypes related to astrocyte dysfunction, subsequent analyses were performed on male mice (Acaz‐Fonseca, Duran, Carrero, Garcia‐Segura, & Arevalo, 2015; Arnold, de Araujo, & Beyer, 2008).

To rule out compensatory effects caused by *Afg3l1* expression, we established an *Afg3l1* ^−/−^: *Afg3l2* ^*fl/fl*^ mouse line, crossed it with the GFAP‐Cre‐ER™ line, and induced *Cre* expression as already described (Figure 1g). At 3 weeks of age, mice bearing astrocyte‐specific deletion of both *Afg3l2* and *Afg3l1* (dubbed astro‐DKO, genotype: *Afg3l1* ^−/−^: *Afg3l2* ^*fl/fl*^: GFAP‐Cre^tg/wt^) were indistinguishable from *Afg3l1* ^−/−^ littermates (abbreviated to L1^−/−^) that we used as control animals. Surprisingly, only 1 week later, astro‐DKO mice showed a dramatic phenotype characterized by failure to gain weight, kyphosis, abnormal posture of the hindlimbs and uncoordinated movements, independent from the gender (Figure 1h,i). We scored these mice for signs of ataxia, using a composite phenotype evaluation scale that included assessment of hind limb clasping, ledge test, gait, and kyphosis (Guyenet et al., 2010). In agreement with their severe phenotype, astro‐DKO mice obtained high scores for all modalities tested (Figure 1j). Given the severity of the phenotype, the mice were euthanized at this age for ethical reasons.

In conclusion, deficiency of *Afg3l2* in postnatal astrocytes triggers a late‐onset motor impairment, while the combined loss of *Afg3l2* and *Afg3l1*, which leads to ablation of the *m‐*AAA protease and mimics the effect of AFG3L2 loss in humans, elicits a dramatic neurological phenotype that develops within days.

### 3.2 Deficiency of the *m‐*AAA protease leads to abnormalities and loss of Bergmann glia

To explore how a deficiency of *m‐*AAA protease subunits affects astrocytes, we restricted our investigations to the cerebellum, which is the tissue most affected in human neurological disorders caused by *AFG3L2* mutations. Cerebellar astrocytes and in particular BG are efficiently targeted after tamoxifen injection, as demonstrated by crossing the GFAP‐Cre‐ER™ transgenic line with reporter lines (Chow et al., 2008; Figure S1A,B). Moreover, functional interactions between BG and PCs in the cerebellum have been extensively studied (Bellamy, 2006; De Zeeuw & Hoogland, 2015). This analysis also allows us to directly compare phenotypes of astrocyte‐specific or neuronal‐specific deficiency of *Afg3l2* in the same brain area, because we have previously characterized a PC‐specific *Afg3l2* knock‐out line (Almajan et al., 2012).

**Figure 2:**
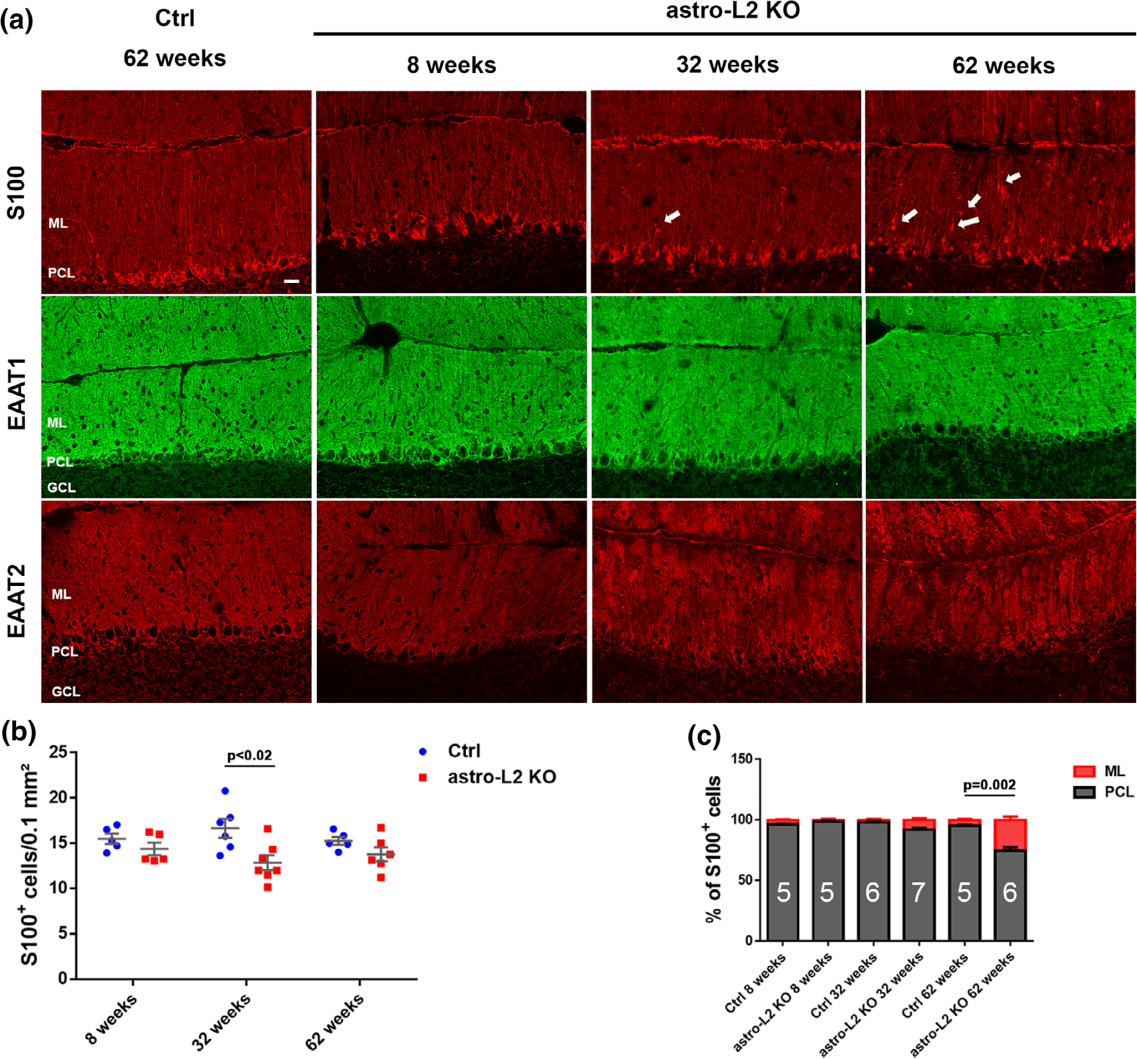
Astrocyte‐specific *Afg3l2* deletion causes BG degenerative changes. (a) Immunostaining of BG using S100, EAAT1, or EAAT2 antibodies at different time‐points (*n* at least three for each group at each time‐point). Scale bar: 20 μm. Arrows indicate displaced BG cell bodies in ML. (b) Dot plots showing quantification of S100^+^ cell bodies in the PCL and ML at different ages (mean ± *SEM* is indicated). (c) Distribution of S100^+^ cells in the PCL and ML at different ages (*n* is indicated in each column). *p* values were obtained by Student's *t* test. BG, Bergmann glia; GCL, granule cell layer; ML, molecular layer; PCL, PC layer [Color figure can be viewed at wileyonlinelibrary.com]

Nissl stainings did not show gross alterations in the layer organization of the cerebellum of astro‐L2 KO and astro‐DKO mice (Figure S3). To assess the number, morphology, and position of the BG, we performed immunofluorescence analysis using several astrocytic markers on cerebellar sections at different time points in both mouse models. In control mice of any age and in 8‐week‐old astro‐L2 KO mice, S100 staining decorated BG cell bodies located in the PCL and their radial projections extending to the pial surface (Figure 2a; Figure S4). At 32 weeks of age, BG in astro‐L2 KO mice appeared less organized, showing a retraction of the radial processes that failed to fully extend to the pial surface and the displacement of a few cell bodies from their original position around the PC cell body into the ML (Figure 2a). This phenotype was more pronounced at 62 weeks, when in several areas the radial processes of BG did not reach the most distal part of the ML and appeared stunted (Figure 2a). Quantification of S100^+^ BG cell bodies located in the PCL and ML did not show prominent cell loss, but confirmed BG dislocation in the ML (Figure 2b,c). We also assessed the expression of glutamate transporters, which are crucial for astrocytic function. EAAT2 showed an abnormal patchy expression pattern in BG of astro‐L2 KO, at both 32 and 62 weeks (Figure 2a; Figure S4). Surprisingly, EAAT1 expression was not overtly affected (Figure 2a; Figure S4). These data suggest that BG are not only abnormal in morphology, but also remodel their gene expression profile.

**Figure 3:**
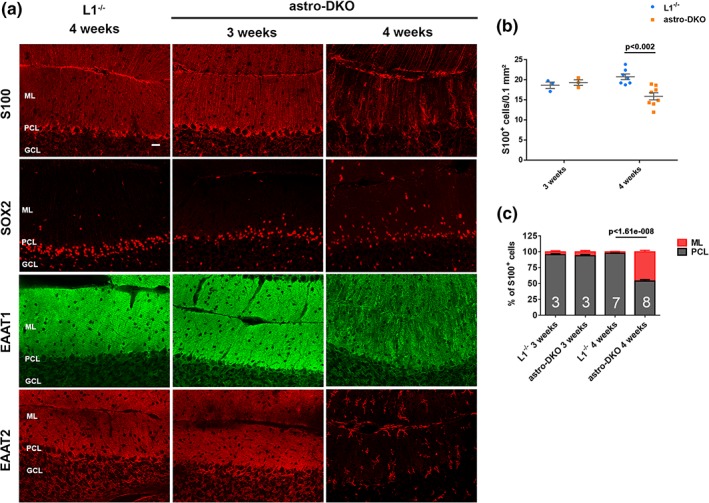
*m*‐AAA protease ablation in astrocytes induces acute BG abnormalities and death of targeted cells. (a) Immunostaining of astrocytes using S100, SOX2, EAAT1, or EATT2 antibodies at the indicated time‐points (*n* ≥ 3 for each group at each time‐point). Scale bar: 20 μm. (b) Dot plots showing quantification of S100^+^ cells in the PCL and ML at 3 and 4 weeks of age (mean ± *SEM* is indicated). (c) Distribution of S100^+^ cells in the PCL and ML at 3 and 4 weeks of age (*n* is indicated in each column). *p* values were obtained by Student's *t* test. BG, Bergmann glia; ML, molecular layer; PCL, PC layer [Color figure can be viewed at wileyonlinelibrary.com]

The BG phenotype observed upon deficiency of *Afg3l2* was severely exacerbated in the astro‐DKO mice. These mice developed pronounced and acute BG morphological alterations between 3 and 4 weeks of age (Figure 3a). While at 3 weeks S100, SOX2, EAAT1, and EAAT2 stainings appeared indistinguishable from L1^−/−^ littermates, at 4 weeks S100‐labeled cell bodies were prominently displaced from their position and migrated into the ML, while their radial processes retracted and failed to reach the uppermost part of the ML (Figure 3a,c). The number of S100^+^ cell bodies located in the PCL and ML was also reduced (Figure 3b). EAAT2 expression was almost completely lost, while EAAT1 was drastically downregulated at 4 weeks (Figure 3a). Remarkably, in the astro‐DKO the EAAT2 antibody stained cells that were morphologically highly reminiscent of activated microglia, as confirmed by double staining with the Iba1 antibody (Figure S5A).

We conclude that deficiency of *m*‐AAA protease subunits alters the morphology and the gene expression profile of BG and ultimately causes the loss of S100^+^ astrocytes. The age of onset and progression of this phenotype depends on the amounts of residual subunits of the *m*‐AAA protease, indicating that *Afg3l1* largely compensates for *Afg3l2* function in these cells.

### 3.3 *m‐*AAA protease deficiency in astrocytes causes mitochondrial morphological abnormalities but no overt respiratory deficit

**Figure 4:**
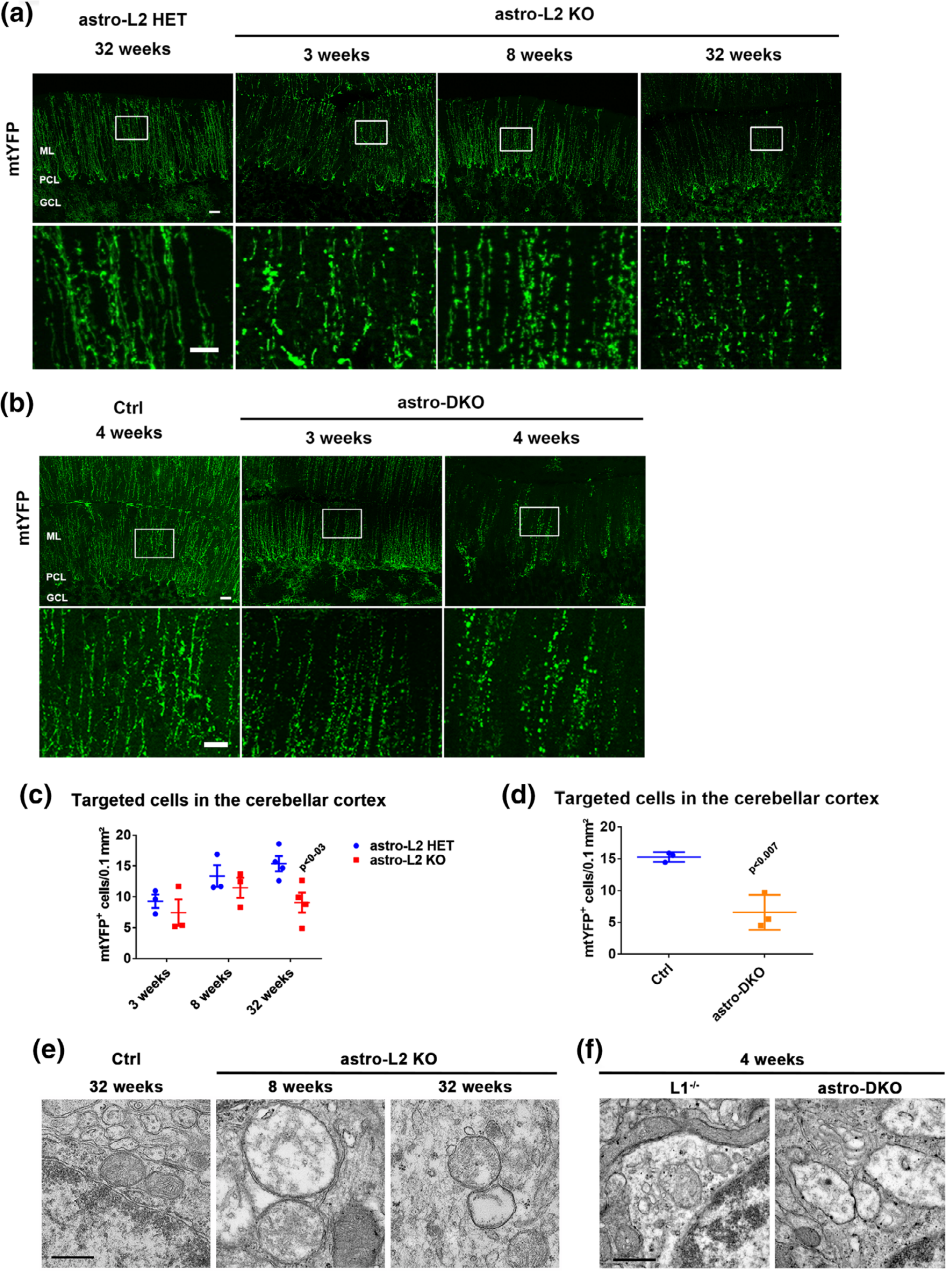
Mitochondrial fragmentation and ultrastructural abnormalities in BG of astro‐L2 KO and astro‐DKO mice. (a) Endogenous mtYFP fluorescent signal reveals targeted cells at different ages (genotypes: astro‐L2 HET = mtYFP^tg/+^:*Afg3l2* ^*fl/+*^:GFAP‐Cre^tg/wt^; astro‐L2KO = mtYFP^tg/+^:*Afg3l2* ^*fl/fl*^:GFAP‐Cre^tg/wt^). Scale bar: 20 μm; scale bar enlargement: 10 μm. (b) Endogenous mtYFP fluorescent signal reveals targeted cells at different ages (genotypes: Ctrl = mtYFP^tg/+:^GFAP‐Cre^tg/wt^; astro‐DKO: mtYFP^tg/+:^ *Afg3l1* ^−/−^:*Afg3l2* ^*fl/fl*^:GFAP‐Cre^tg/wt^). Scale bar: 20 μm; scale bar enlargement: 10 μm. (c) Quantification of mtYFP^+^ cells in the PCL at different ages in astro‐L2 HET and astro‐L2 KO mice. (d) Quantification of mtYFP^+^ cells in the PCL in Ctrl and astro‐DKO mice. (e, f) Ultrastructural analysis of mitochondrial morphology in BG. Scale bar: 500 nm. In all graphs, individual data point, and means ± *SEM* are indicated. *p* values were obtained by Student's *t* test. BG, Bergmann glia; GFAP, glial fibrillary acidic protein; PCL, PC layer [Color figure can be viewed at wileyonlinelibrary.com]

To unravel why loss of the *m*‐AAA protease affects BG, we first explored mitochondrial morphology, which is known to be affected in neurons lacking *Afg3l2* (Almajan et al., 2012). To recognize mitochondria of BG deleted for *Afg3l2*, we crossed the mice with the mtYFP reporter line (Sterky et al., 2011). Surprisingly, we observed fragmentation of mitochondria in radial BG projection in both astro‐L2 KO and astro‐DKO mice already at 3 weeks of age (Figure 4a,b), well before the appearance of BG morphological abnormalities. Furthermore, we could quantify the number of targeted BG at different ages, with the caveat that accumulation of the mtYFP reporter requires residual mitochondrial membrane potential. Of note, the number of mtYFP‐positive BG slightly increased at 8 weeks compared to 3 weeks, possibly reflecting postnatal proliferation of BG (Shiga, Ichikawa, & Hirata, 1983; Figure 4c). The number of targeted BG in the PCL highlighted by the mtYFP endogenous fluorescence signal was similar in astro‐L2 KO mice and Ctrl littermates at 3 and 8 weeks, but decreased at 32 weeks of age, in agreement with the data obtained with the S100 antibody (Figure 4c). Similarly, the number of mtYFP‐positive BG in the PCL was significantly lower in the astro‐DKO compared to *Afg3l1* ^−/−^ littermates at 4 weeks of age (Figure 4d).

We also examined mitochondrial ultrastructure by electron microscopy (Figure 4e,f). In both models, mitochondria in the cell body and the radial processes of BG showed signs of swelling and had a reduced number of cristae, very similarly to what was already observed in neurons or in oligodendrocytes deleted for subunits of the *m‐*AAA protease (Almajan et al., 2012; Wang et al., 2016). To investigate if these structurally abnormal mitochondria lead to respiratory deficiency, we performed combined COX‐SDH stainings on cerebellar sections at different time points in both models. Surprisingly, we could not detect evidence of COX deficiency in astro‐L2 KO or astro‐DKO mice (Figure S6). Our data thus uncouple astrocyte demise from respiratory dysfunction.

### 3.4 Deficiency of the *m‐*AAA protease in astrocytes induces inflammation and metabolic stress responses

**Figure 5:**
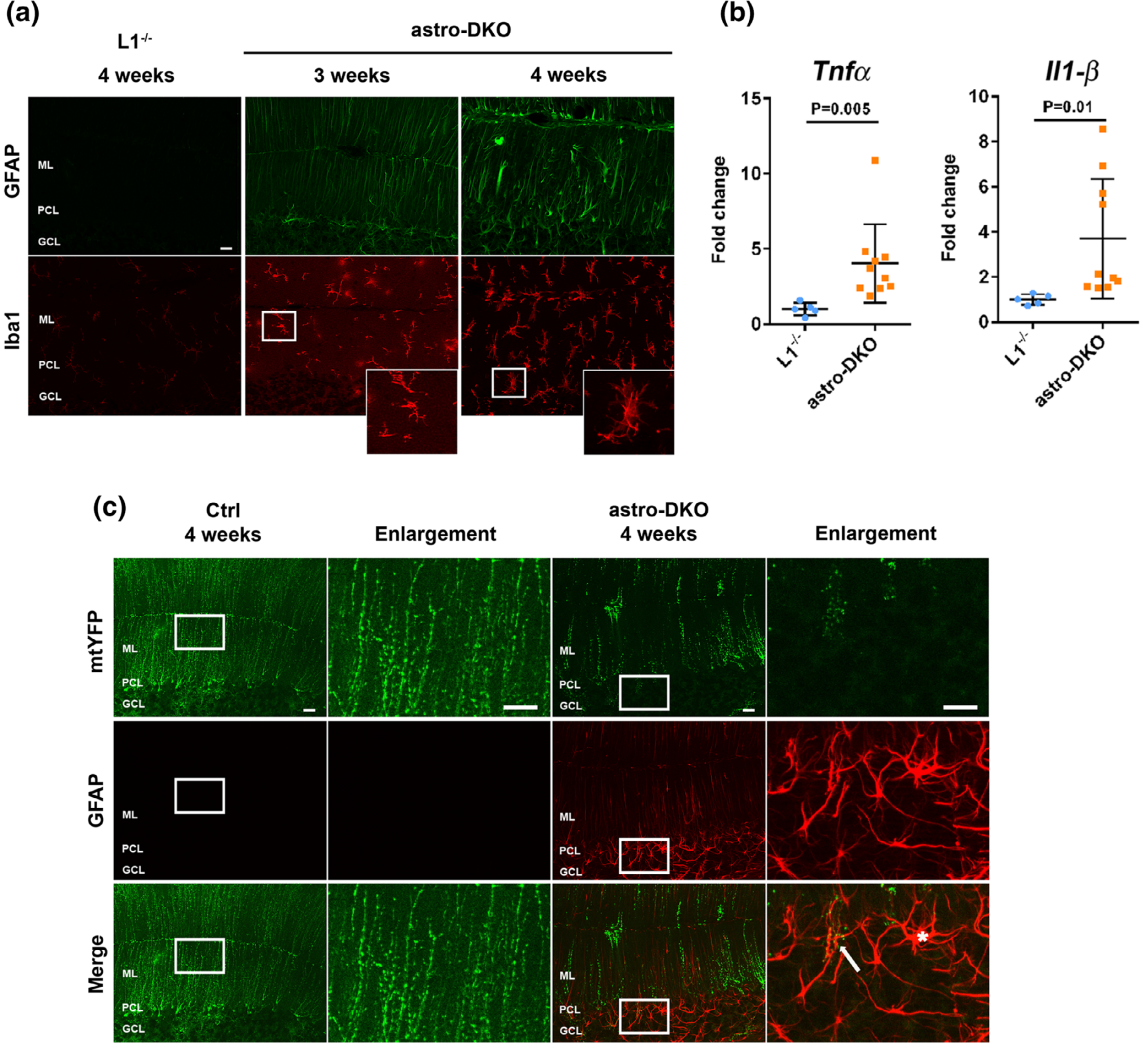
Deficiency of the *m*‐AAA protease in astrocytes induces reactive astrogliosis and inflammation. (a) Immunostaining for GFAP and Iba1 at 3 and 4 weeks show reactive astrogliosis and microglia activation in astro‐DKOs (*n* = 3 at each time‐point for each group). (b) mRNA quantification of inflammatory markers at 4 weeks. Data are visualized as dot plots, including mean ± *SD. p* values were obtained by Student's *t* test. (c) Immunostaining of GFAP and mtYFP in 4 weeks old Ctrl and astro‐DKO (*n* = 3 at each time‐point for each group). Scale bars =20 μm. GFAP, glial fibrillary acidic protein [Color figure can be viewed at wileyonlinelibrary.com]

In the central nervous system, reactive astrogliosis and microglia activation are often observed upon pathological insults that involve tissue damage, and may contribute to pathology in a non‐cell‐autonomous manner (Hanisch & Kettenmann, 2007; Liddelow et al., 2017). Indeed, astro‐L2 KO mice showed signs of reactive astrogliosis detected by upregulation of GFAP at 32 weeks, but not at 62 weeks, possibly reflecting a transient response concomitant with the peak of cell demise (Figure S5B). Microglia was not activated in the cerebellum of these mice, confirming that the inflammatory response is mild and transient (Figure S5B). In contrast, astro‐DKO mice showed pronounced reactive astrogliosis, infiltration of microglia with ameboid morphology (Figure 5a; Figure S5A), and increased expression of the proinflammatory cytokines TNF‐α and IL‐1β at 4 weeks (Figure 5b).

We then asked whether reactive astrogliosis represents a cell‐autonomous response to deficiency of the *m‐*AAA protease or if reactive astrocytes are nontargeted cells that respond to tissue damage. To distinguish targeted from untargeted astrocytes, we investigated mice carrying the mtYFP reporter. In many cases, reactive GFAP‐positive astrocytes in the astro‐L2 KO were also positive for mtYFP, indicating that deletion of *Afg3l2* triggers cell‐autonomously an inflammatory phenotype (Figure S5C). In contrast, in the astro‐DKOs a mixture of reactive astrocytes positive and negative for the mtYFP signal were detected at 4 weeks (Figure 5c). Based on these findings, we hypothesize that deficiency of the *m‐*AAA protease induces a reactive phenotype in a cell‐autonomous fashion, which is then amplified by recruiting other inflammatory cells, including nontargeted reactive astrocytes and microglia.

**Figure 6:**
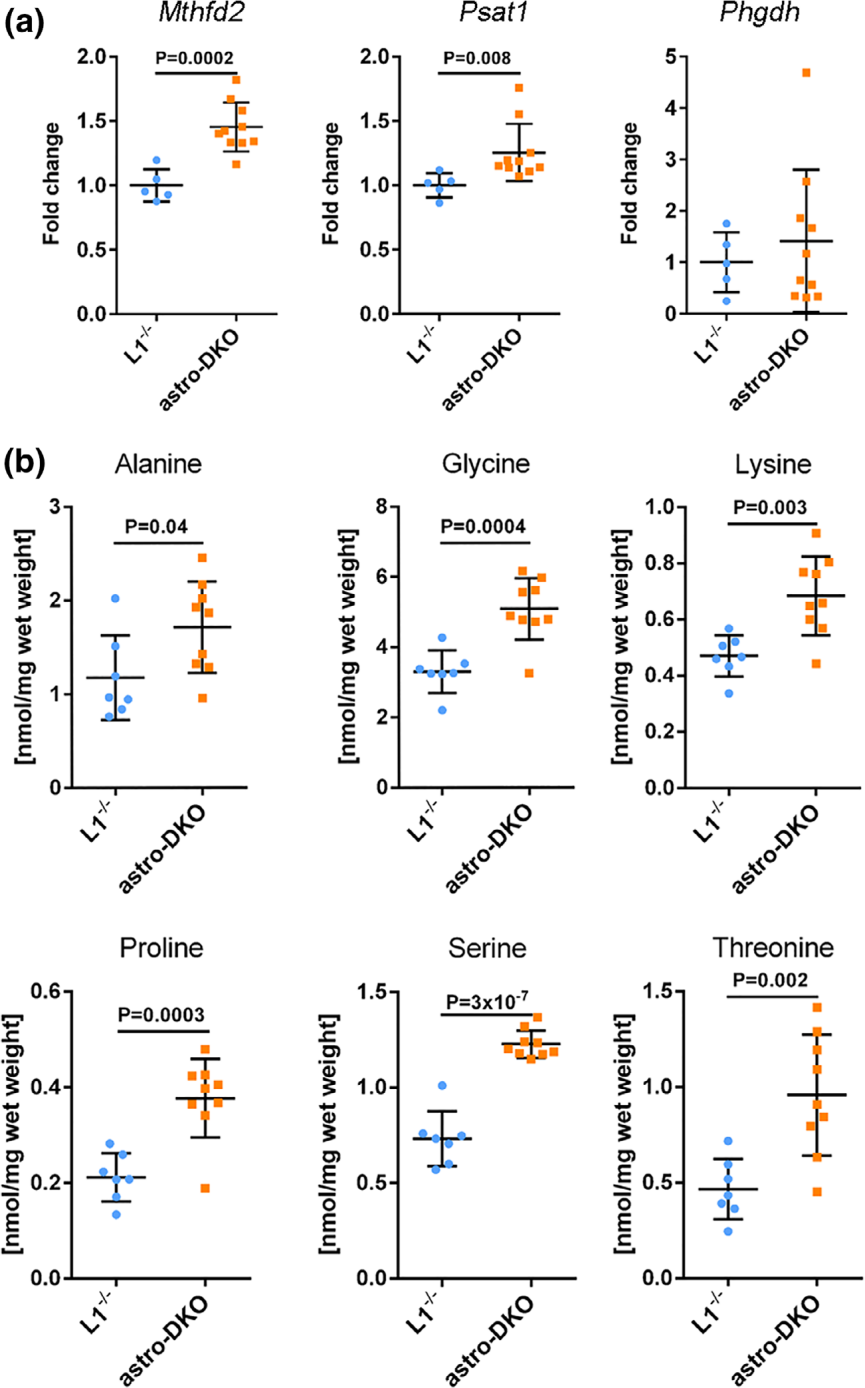
*m*‐AAA protease deficiency in astrocytes leads to metabolic stress responses. (a) mRNA levels of genes involved in folate cycle and serine biosynthesis in astro‐DKO and L1^−/−^ mice, visualized as individual data points, mean and *SD*. (b) Amino acid levels in cerebellar lysates from astro‐DKO and L1^−/−^ mice at 4 weeks, visualized as dot plots, including mean ± *SD. p* values were obtained by Student's *t* test [Color figure can be viewed at wileyonlinelibrary.com]

Recent data indicate that to fully comprehend the phenotypic manifestations of mitochondrial diseases, it is crucial to take into account metabolic stress responses of the affected cells (Khan et al., 2017; Nikkanen et al., 2016; Restelli et al., 2018; Suomalainen & Battersby, 2018). A common feature observed in mouse models of mitochondrial diseases characterized by defects in mtDNA gene expression is the upregulation of the mitochondrial folate cycle and the serine biosynthesis pathway (Kuhl et al., 2017; Nikkanen et al., 2016). To investigate if these pathways are activated also upon *m‐*AAA protease deficiency in astrocytes, we analyzed the expression of *Mthfd2*, encoding for the rate‐limiting enzyme of the mitochondrial folate cycle, and of *Phgdh* and *Psat1*, involved in serine biosynthesis, in total cerebellar lysates of astro‐DKO mice. *Mthfd*2 and *Psat1* were clearly upregulated at the mRNA level at 4 weeks, demonstrating a metabolic stress response in the cerebellum of the astro‐DKO mice (Figure 6a). To substantiate these findings, we measured amino acid levels. Consistent with previous observations (Nikkanen et al., 2016), we detected an increase in the level of alanine, glycine, lysine, proline, serine, and threonine at 4 weeks of age (Figure 6b; Figure S7).

Altogether, these findings indicate that astrocyte demise is associated with the acquisition of a reactive state, and a metabolic remodeling to enhance folate and serine biosynthesis.

### 3.5 *m‐*AAA protease deficiency in astrocytes leads to morphological degeneration and alterations in intrinsic electrophysiological properties of Purkinje cells

**Figure 7:**
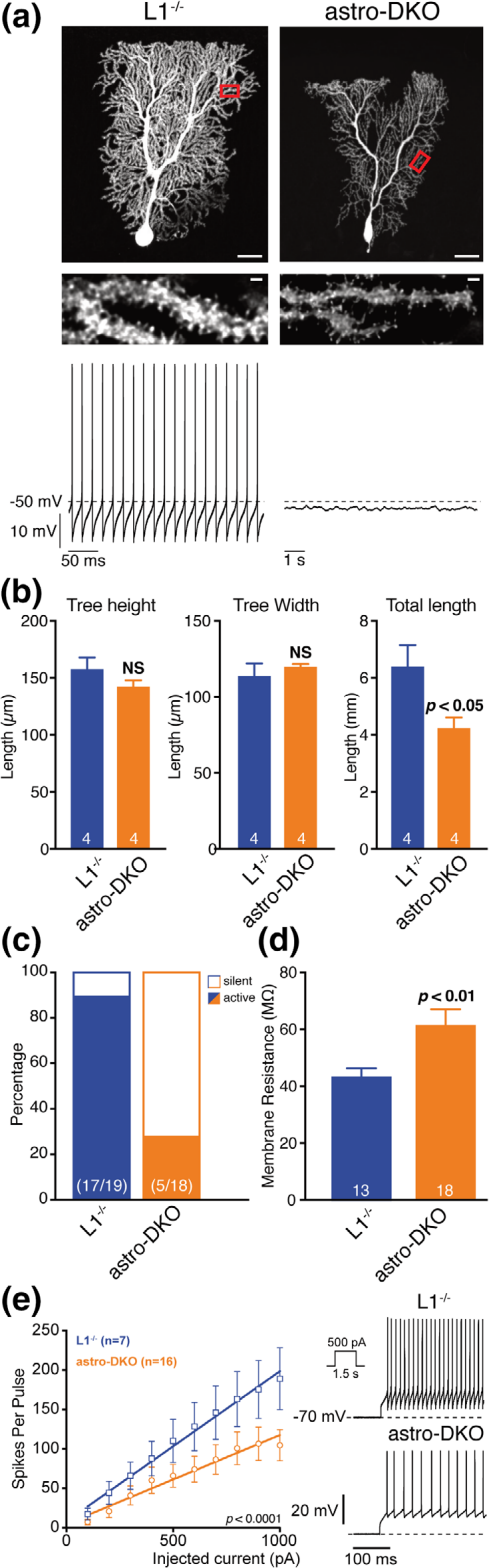
Morphology and intrinsic electrophysiological properties of Purkinje cells are changed in astro‐DKO mice. (a) Top: Images of single biocytin‐streptavidin labeled PCs of L1^−/−^ and astro‐DKO mice and enlarged view of the boxed dendritic branches. Scale bars: 20 μm; inset: 1 μm. Bottom: Original recordings of the corresponding spontaneous activity. (b) Analysis of total dendritic length of single biocytin‐streptavidin labeled PCs of L1^−/−^ and astro‐DKO mice with similar dendritic tree width and height. *p* and *n* values are given in the graphs (dendritic tree width and length, two‐tailed unpaired *t* test; total dendritic length, two‐tailed Mann–Whitney test). (c) Percentage of PCs that generated typical spontaneous activity (solid bar) and PCs that were silent (empty bar) in L1^−/−^ and astro‐DKO mice. *n* in bars indicate the number of neurons analyzed (from 3 to 5 mice per genotype). (d) Mean input resistance of L1^−/−^ and astro‐DKO mice (*n* in bars indicate the number of neurons tested). *p* values are given in the graphs (two‐tailed Welch's *t* test). (e) Excitability of L1^−/−^ and astro‐DKO mice. Left: Mean spike counts during current pulses (1.5 s) as a function of injected current from a holding potential of −70 mV. Right: Representative voltage responses (initial 300 ms) of PCs to 500 pA depolarizing current pulses. Mean values are represented as mean ± *SEM. p* values are given in the graphs, *F* test. PC, Purkinje cells [Color figure can be viewed at wileyonlinelibrary.com]

We then asked if PCs, which are the main output inhibitory neurons from the cerebellum, may be secondarily affected. PCs are orderly aligned next to each other in the PCL and project an elaborate dendritic tree into the ML to form synaptic contacts with axons of granule cells, the parallel fibers. Synapses between PCs and parallel fibers are glutamatergic and are enwrapped by BG. Both astro‐L2 KO and astro‐DKO mice showed morphological alterations of PCs (Figure 7a; Figure S8A,B). In the astro‐L2 KO mice, the regular distribution of PCs in one single layer was perturbed at 32 weeks, and even more at 62 weeks (Figure S8A). PCs appeared unusually packed next to each other in some areas, while in other regions the cell bodies moved away from the PC layer and migrated in the molecular layer. The dendritic arborization of PCs also appeared less pronounced and organized (Figure S8A). These morphological phenotypes of PCs were even more prominent in astro‐DKO mice (Figure 7a; Figure S8B). Analyzing single biocytin‐streptavidin labeled PCs of L1^−/−^ and astro‐DKO mice with similar dendritic tree width and height, showed that the total dendritic length was reduced by ~30% in PCs of astro‐DKO mice (*p* = .0286, two‐tailed Mann–Whitney test; Figure 7b).

Next, we assessed whether the observed morphological changes of PCs in astro‐DKO mice affect the intrinsic electrophysiological properties of these neurons. To this end, we performed current clamp recordings from PCs in brain slices of astro‐DKO mice and L1^−/−^ littermates. To conserve the integrity of the cytosolic pathways the recordings were performed in the perforated patch clamp configuration. For post hoc morphological analysis, cells were loaded with biocytin by converting the perforated patch configuration to the whole cell configuration at the end of the recording. During the recording, GABAergic and glutamatergic synaptic input was pharmacologically blocked to minimize influence from the network. Assessment of several key electrophysiological parameters such as spontaneous firing rate, input resistance, and excitability revealed significant differences between PCs of L1^−/−^ and astro‐DKO mice (Figure 7c,e)). Overall, PCs of astro‐DKO mice were less excitable than those of L1^−/−^ mice. In the latter ~90% (17/19) of the PCs generated typical spontaneous activity, while only ~28% (5/18) of PCs in astro‐DKO mice generated spontaneous action potentials (Figure 7c). Note that the spontaneously silent neurons still generated action potentials upon depolarizing current injection. However, the relative firing rate was lower than in control PCs, which is reflected in the shallower slope of the current‐frequency plots (*p* < .0001, *F* test; Figure 7e). Interestingly, the decreased excitability of PCs in astro‐KO mice was accompanied by a significant increase in cell input resistance (*p* = .0038, two‐tailed Welch's *t* test; Figure 7d), which indicates a change in the ion channel composition and not only a mere decrease in dendritic arborizations (i.e., the cell surface).

### 3.6 Loss of the *m‐*AAA protease in cerebellar astrocytes leads to upregulation of necroptotic markers

**Figure 8:**
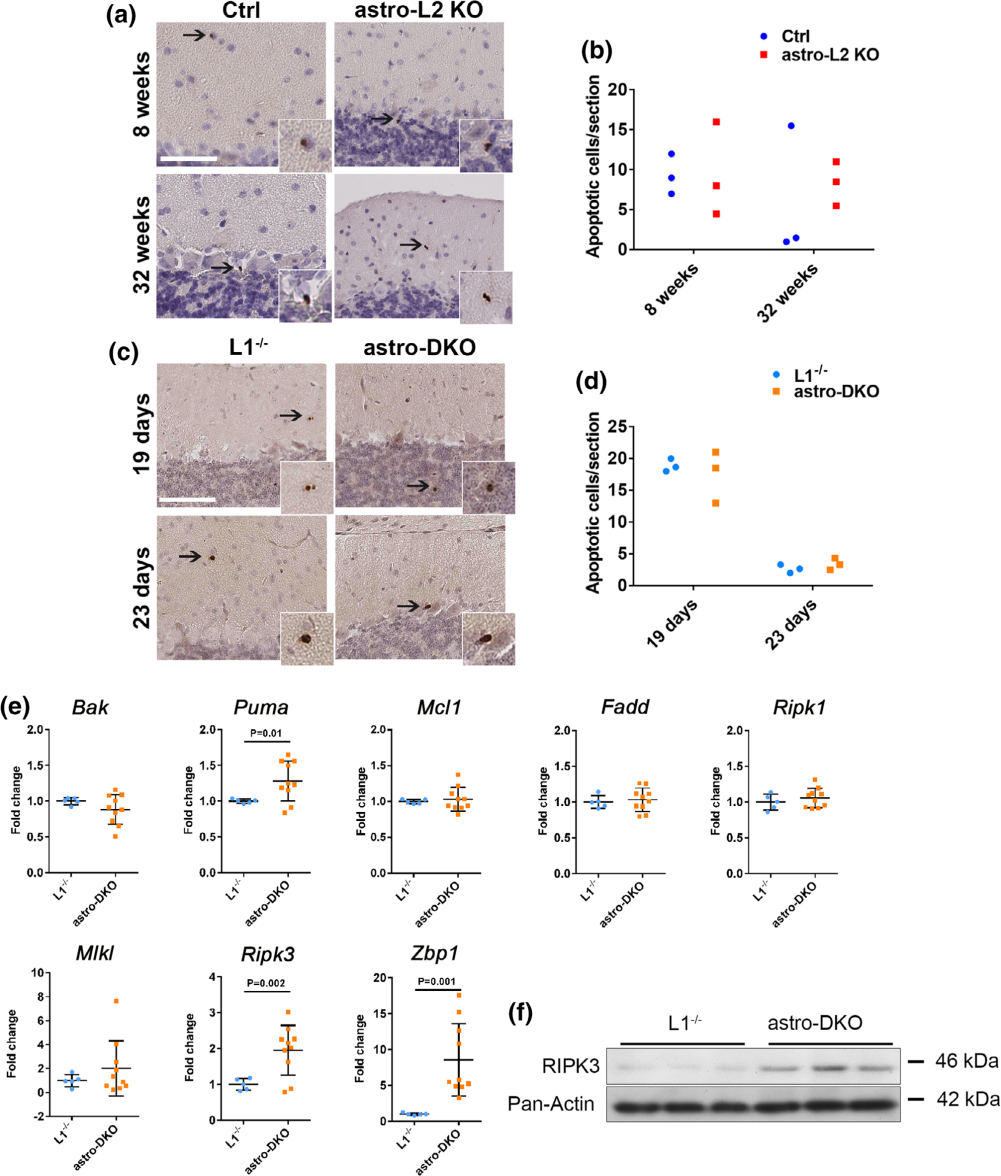
Necroptotic markers are upregulated in the cerebellum upon loss of the *m*‐AAA protease in astrocytes. (a–d) Immunohistochemistry for cleaved caspase 3 and relative quantification in the cerebellum. All positive cells in one section were counted from at least three sections per mouse (*n* = 3 for each group at each time‐point). Data are shown as dot blots. Scale bar: 80 μm. (e) mRNA expression of apoptotic and necroptotic markers in astro‐DKO and L1^−/−^ mice at 4 weeks, visualized as individual data points, mean ± *SD. p* values were obtained by Student's *t* test. (f) Western blot analysis of RIPK3 and pan‐actin as loading control in total cerebellar lysates (50 μg of protein lysate/well) [Color figure can be viewed at wileyonlinelibrary.com]

Given the complex astrocytic and neuronal phenotype in mutant mice, we investigated if specific death pathways are activated in the cerebellum. Immunostainings to reveal cleaved caspase‐3 did not show an increase in the number of apoptotic cells in both astro‐L2 KO and astro‐DKO mouse models (Figure 8a,d). We therefore assessed the expression of several markers for apoptosis and necroptosis in the cerebellum of 4‐week‐old astro‐DKO mice. Interestingly, we found a significant upregulation of *Ripk3*, encoding for a kinase involved in necroptosis, and of *Zbp1*, encoding Z‐DNA binding protein 1, an activator of necroptosis (Figure 8e). In contrast, apoptotic markers were not differentially expressed, with the exception of a mild increase of *Puma* levels (Figure 8e). We confirmed the increase in RIPK3 also by western blot analysis of cerebellar lysates (Figure 8f). Overall, our data indicate the activation of necroptotic pathways in the cerebella of mice carrying astrocyte‐specific deletion of the *m‐*AAA protease.

## 4 DISCUSSION

The *m*‐AAA protease plays crucial roles to maintain mitochondrial homeostasis in neurons, as underlined by the spectrum of neurological diseases associated with mutations in genes encoding the individual subunits SPG7 and AFG3L2 (Casari et al., 1998; Di Bella et al., 2010; Eskandrani et al., 2017; Pierson et al., 2011). Here, we show that astrocyte dysfunction in the cerebellum contributes to the pathogenic cascade. To this end, we have used two different mouse models in which the dosage of the *m*‐AAA protease was reduced to different degrees in astrocytes postnatally. In the astro‐L2 KO, astrocytes still harbored intact *Spg7* and *Afg3l1* genes. The respective proteins can form functional *m*‐AAA protease isoenzymes (Koppen et al., 2007), thus explaining the relative mild and late‐onset behavioral phenotype observed in the astro‐L2 KO mice. As *Afg3l1* is functionally homologous to *Afg3l2* but is a pseudogene in humans (Kremmidiotis et al., 2001), we have also developed the astro‐DKO mouse line. This model more faithfully reflects the genotype of human patients carrying homozygous loss‐of‐function mutations in *Afg3l2* (Eskandrani et al., 2017; Pierson et al., 2011). Surprisingly, astro‐DKO mice developed an acute postnatal phenotype within a week, associated with severe signs of ataxia, kyphosis, and cachexia.

We demonstrate a critical role of the *m*‐AAA protease to maintain the homeostasis of BG, which fully enwrap PCs and their synapses, and regulate development and functional properties of these neurons (Bellamy, 2006; De Zeeuw & Hoogland, 2015; Iino et al., 2001). In both mouse models, despite with different time‐course and severity, targeted BG showed progressive degenerative signs characterized by an ectopic position, a disrupted morphology, and an altered expression of several markers, with downregulation of the calcium‐binding protein S100 and the glutamate transporter EAAT2, and upregulation of GFAP, a signature of a “reactive” phenotype. Moreover, mutant astrocytes displayed an abnormal mitochondrial network, and activated a metabolic stress response. The time‐dependent decrease of BG labeled using a *Cre*‐dependent mtYFP reporter or with S100 antibodies strongly suggests that the end‐point of this degenerative process is the death of targeted astrocytes. This conclusion is also supported by the upregulation of markers of necroptosis in the cerebellum of astro‐DKO mice. Necroptosis is a form of regulated necrotic cell death, in which RIPK1/RIPK3 kinases mediate activation of the MLKL kinase (Pasparakis & Vandenabeele, 2015), and is emerging as a relevant pathway of cell death in other neurodegenerative conditions, such as amyotrophic lateral sclerosis (Ito et al., 2016; Re et al., 2014). However, as the expression of necroptotic markers has been quantified in total cerebellar lysates, we cannot exclude a contribution from activated microglia or from degenerative changes of PCs. Furthermore, it is possible that the expression of the mtYFP reporter is affected in mitochondria lacking the *m*‐AAA protease. Therefore, additional studies will be needed to fully understand if necroptotic cell death follows astrocyte degeneration.

Despite the fact that astrocytes contain a very dynamic and elongated mitochondrial network (Stephen, Gupta‐Agarwal, & Kittler, 2014), only very recently the contribution of astrocytes to mitochondrial diseases has been investigated. Our study represents in fact one of the few in which a mitochondrial protein has been deleted specifically in murine astrocytes. Remarkably, the outcomes of these genetic manipulations have been rather different. Astrocyte‐specific deletion of *Cox10*, a complex IV assembly factor, despite leading to complex IV deficiency and a respiratory deficit, caused neither cell death nor secondary neuronal degeneration, but rather increased the glycolytic metabolism of the cells (Supplie et al., 2017). In contrast, inactivation of the helicase Twinkle specifically in astrocytes, leading to OXPHOS deficiency and loss of mtDNA, induced a chronic activation of astrocytes, causing spongiosis, loss of neurons, and inflammation (Ignatenko et al., 2018). In this study, we did not find evidence of respiratory deficiency, but also observed cell‐autonomous activation of targeted astrocytes and infiltration of microglia. Of interest is also the high upregulation of *Zbp1* in the cerebellum of astro‐DKO mice. A part from its role as an innate immune sensor of both RNA and DNA viruses (Kuriakose & Kanneganti, 2018), ZBP1 is a critical mediator of necroptosis‐induced inflammation in the skin (Lin et al., 2016), raising the possibility of its implication in reactive astrogliosis.

What is the link between mitochondrial dysfunction and the inflammatory phenotype of astrocytes? The lesson from these distinct mouse models is that it is unlikely that OXPHOS deficiency per se plays a role. Instead, we favor the hypothesis that the metabolic remodeling associated with the mitochondrial stress response may induce the astrocyte “reactive” phenotype, which ultimately is the culprit of the secondary neuronal degeneration. While at present only a speculation, some support in this direction comes from the finding that *Mthfd2* is upregulated in reactive astrocytes induced by transient middle cerebral artery occlusion in vivo (Zamanian et al., 2012) or by exposure to IL‐1β in vitro (Teh et al., 2017). Future studies will be needed to fully understand how mitochondrial metabolism can influence reactive astrogliosis, and whether this cell‐autonomous response has a damaging or protective role (Liddelow et al., 2017).

Both this study and the work of Ignatenko et al. (2018) highlight how mitochondrial dysfunction in astrocytes can have downstream effects on neuronal function and survival. We show here that decreasing the levels of the *m*‐AAA protease in BG is sufficient to cause non‐cell‐autonomous morphological degeneration and to change the electrophysiological properties of PCs. Although in the astro‐DKO mice we cannot formally exclude that lack of *Afg3l1* in PCs also contributes to the phenotype, we demonstrate degenerative and electrophysiological abnormalities of these neurons that are not detected in the single L1^−/−^ model. Most PCs in the astro‐DKO mice have a reduced dendritic arborization, lack spontaneous firing activity, and are less excitable in response to depolarizing current injection, despite having an increased cell input resistance. These changes could simply be a predegenerative phenotype due to lack of metabolic support from the BG, however they may also reflect compensatory changes in PCs to cope with increased glutamatergic excitation. A crucial task of BG in fact is to maintain a low concentration of glutamate in the synaptic cleft, by the action of the glutamate–aspartate transporter EAAT1 and the Na^+^‐dependent glutamate transporter EAAT2, which is especially downregulated in both astro‐L2 KO and astro‐DKO mice. Notably, in the astro‐DKO, microglia upregulates EAAT2 expression, probably as a compensatory mechanism. Impaired glutamate transport by BG as a cause of PC degeneration has been already seen in other mouse models. BG‐restricted expression of ataxin‐7 carrying a polyglutamine expansion was sufficient to cause neurodegeneration, owing to reduced expression and function of the EAAT1 glutamate transporter (Custer et al., 2006). Moreover, in a mouse model of myotonic dystrophy, BG and not neurons appeared to be the preferential target of RNA toxicity in the cerebellum, leading to EAAT2 downregulation, increased glutamate neurotoxicity, and PC firing abnormalities (Sicot et al., 2017).One of the most accredited pathogenic hypotheses in cerebellar ataxias, including SCA28, explains the degeneration of PCs with excitotoxicity, caused by excessive glutamatergic stimulation. Excitotoxicity leads to dark cell degeneration, a specialized form of calpain‐dependent cell death. Dark cell degeneration of PCs has been indeed observed in different mouse models with *Afg3l2* deficiency (Maltecca et al., 2008, 2009). So far, cell‐autonomous abnormal Ca^2+^ homeostasis has been proposed as the main mechanism for calpain activation in PCs lacking AFG3L2. Maltecca et al. (2012) showed a decreased ability of AFG3L2‐deficient mitochondria to buffer Ca^2+^, while we demonstrated an increased assembly of the mitochondrial Ca^2+^ uniporter MCU upon *m*‐AAA protease deficiency, possibly leading to increased calcium uptake, and opening of the mitochondrial permeability transition pore (König et al., 2016). Both mechanisms strongly rationalize the degeneration of PCs as a consequence to increased Ca^2+^ influx upon glutamatergic stimulation. In agreement with this pathogenic hypothesis, two different interventions to limit Ca^2+^ entry into PCs improved the neurological phenotype of full‐body *Afg3l2* heterozygous mice (Maltecca et al., 2015). One of these strategies entailed increasing the expression of the astrocyte glutamate transporter EAAT2 with the antibiotic ceftriaxone (Maltecca et al., 2015; Rothstein et al., 2005). Our data indicate that BG dysfunction may magnify or even cause excitotoxicity, and provide an additional explanation for the therapeutic benefit of ceftriaxone treatment, which may rescue the defective EAAT2 levels and limit BG dysfunction. Notably, the cerebellar phenotype of myotonic dystrophy mice was also ameliorated by ceftriaxone treatment (Sicot et al., 2017).

In conclusion, we propose a revised model for the pathogenesis of neurological diseases linked to *Afg3l2* deficiency, in which astrocytes are not innocent bystanders, but they actively amplify and precipitate neuronal damage, by assuming a reactive status and impairing glutamate uptake. Our work provides additional support to a crucial role of BG dysfunction in ataxic phenotypes, and to the potential benefit of therapies that specifically target this dysfunction.

## CONFLICT OF INTEREST

The authors declare no conflict of interest.

## Supporting information


**Appendix S1:** Supplementary Materials and Methods

**Appendix S2:** Supplementary material for the Reviewers
