# Supplementary material for: Astrocyte‐specific deletion of the mitochondrial m‐AAA protease reveals glial contribution to neurodegeneration
Source: Glia. 2019 Apr 16;67(8):1526–41. doi: 10.1002/glia.23626 (PMC6618114; doi:10.1002/glia.23626)
Supplement: Supplementary file 1 — Appendix S1: Supplementary Materials and Methods [file GLIA-67-1526-s001.docx]

**Supplementary Materials and Methods**

**Real-Time PCR primers**

The following primers were used: HPRT1 forward 5´-TCCTCCTCAGACCGCTTTT-3´, reverse 5´-CATAACCTGGTTCATCGC-3´; TNFα forward 5’-CTTCTGTCTACTGAACTTCGGG-3’, reverse 5’-CAGGCTTGTCACTCGAATTTTG-3’; IL-1β forward 5’-GAAATGCCACCTTTTGACAGTG-3’, reverse 5’-TGGATGCTCTCATCAGGACAG-3’; IL-6 forward 5’-TAGTCCTTCCTACCCCAATTTCC-3’, and reverse 5’-TTGGTCCTTAGCCACTCCTTC-3’; PSAT1 forward 5´- AGTGGAGCGCCAGAATAGAA-3´, and reverse 5´- CTTCGGTTGTGACAGCGTTA-3´; PHGDH forward 5´- GACCCCATCATCTCTCCTGA-3´, reverse 5´- GCACACCTTTCTTGCACTGA-3´; MTHFD2 forward 5´-CTGAAGTGGGAATCAACAGTGAG-3´, and reverse 5´- GTCAGGAGAAACGGCATTGC-3´; FADD forward 5´-CGCCGACACGATCTACTG-3´, and reverse 5´-GGCCAGTCTTTTCCAGTCTC-3´; RIPK1 forward 5´-GGAAGGATAATCGTGGAGGC-3´, and reverse 5´-AAGGAAGCCACACCAAGATC3´; RIPK3 forward 5´-ACCCCACCGAATCCAATG-3´, and reverse 5´-AGTTCCCAATCTGCACTTCAG-3´; MLKL forward 5´-ACTGTGAACTTGGAACCCTG-3´, and reverse 5´-TGCTGATGTTTCTGTGGAGTG-3´; ZBP1 forward 5´-TGTTGACTTGAGCACAGGAG-3´, reverse 5´-TTCAGGCGGTAAAGGACTTG-3´; BAK forward 5´-TGATATTAACCGGCGCTACG-3´, and reverse 5´-AGCTGATGCCACTCTTAAATAGG-3´; PUMA forward 5´-CAGGAAGATCGGAGACAAAGTG-3´, and reverse 5´-AGCACACTCGTCCTTCAAG-3´; MCL1 forward 5´-TTGTAAGGACGAAACGGGAC-3´, and reverse 5´-TCTAGGTCCTGTACGTGGAAG-3´.

**PCR on genomic DNA**

The deletion caused by *Cre* activation (Afg3l2 Δ) was detected on genomic DNA extracted from tail, liver, forebrain and cerebellum of 8-week-old mouse using PCR with the following primers: Afg3l2 forward 5´-TTGGTTTTGTATGTGTTAGGTCAG-3´; and Afg3l2 Δ reverse 5´-ATTTGGAGCTGCGGGTTAG-3´.

**Nissl staining**

Paraffin sections underwent deparaffinization. Sections were dried at RT, and then were immersed in Nissl solution (36 mM Sodium hydroxide, 0.24% acetic acid, 0.25% thionine acetate) for 30-45 sec. The slides were washed in water, then dehydrated in ethanol (gradient 50% to 100%). Finally, the slides were immersed in xylene for 4 minutes and mounted with Eukitt mounting medium. Pictures were acquired using the Axio-Imager M2 microscope equipped with Apotome 2 (Zeiss) and processed using the software AxioVision SE64 Rel. 4.9.1.

**COX-SDH histochemistry**

Following cervical dislocation, cerebella were immersed in 15% sucrose for 4 h and in 30% sucrose O/N at 4°C. The tissue was then embedded in OCT (Tissue-Tek) and frozen on dry ice. 10 µm sections were cut using a cryostat (CM1850, Leica). The sections were then incubated in COX-solution containing 5 mM DAB (Sigma-Aldrich), 500 µM cytochrome C (Sigma-Aldrich) and 2 mg/ml of bovine catalase (Sigma-Aldrich) for 1 h at 37°C. After washing in PBS, sections were then incubated for 1 h at 37°C in SDH-solution containing 1.875 mM NBT (Sigma-Aldrich), 1.3 M sodium succinate (Sigma-Aldrich), 2 mM PMS (Sigma-Aldrich) and 100 mM sodium azide (Sigma-Aldrich). The slides were then dehydrated in ethanol, washed in xylol and mounted in Eukitt (Sigma).

**Amino acid quantification**

Amino acid levels in mouse cerebellum were determined by Liquid Chromatography coupled to Electrospray Ionization Tandem Mass Spectrometry (LC-ESI-MS/MS). Mouse cerebellum samples were thawed on ice and weighed. After addition of ice-cold acetonitrile/0.3 % formic acid 1:1 (v/v) (10 µl/mg tissue), the tissue was homogenized using the Precellys 24 Homogenisator (Peqlab) at 6,500 rpm for 30 sec and directly put on ice again. The protein content of the homogenate was routinely determined using bicinchoninic acid. To 100 µl of homogenate 10 µl of internal standard mixture, containing isotope-labeled amino acids (Sigma) at concentrations of 200 µM, were added. After the addition of 800 µl of methanol, the sample mixtures were vigorously vortexed. The samples were centrifuged (12,000 × g, 5 min, 4 °C) to precipitate proteins. 100 µl of the supernatant were transferred to autoinjector vials and dried under a stream of nitrogen. Amino acids in the dried extracts were derivatized with 6-aminoquinolyl-N-hydroxysuccinimidyl carbamate using the AccQ-Tag Ultra Derivatization Kit (application note 720005189EN, Waters). After derivatization, the sample volume was 100 µl.

LC-MS/MS analysis was performed using a Core-Shell Kinetex C18 column (150 mm × 2.1 mm ID, 2.6 µm particle size, 100 Å pore size, Phenomenex) with detection using a QTRAP 6500 triple quadrupole/linear ion trap mass spectrometer (SCIEX). The LC (1260 Infinity Binary LC System, Agilent) was operated at 40 °C and at a flow rate of 0.4 ml/min with a mobile phase of water with 0.1 % formic acid (solvent A) and acetonitrile with 0.1 % formic acid (solvent B). Prior to sample injection, the column was equilibrated for 1.0 min with 3 % B. After injection of 1 µl of sample, 3 % B was continued for 1.6 min, then linearly changed to 33 % B in 8.2 min, followed by a 0.1 min-linear gradient to 95 % B, which was maintained for 5.3 min and finally restored to 3 % B by a 0.1 min-linear gradient and held for 3.1 min to re-equilibrate the column. The total run time was 18.4 min. Derivatized amino acids were monitored in the positive ion mode with their specific Multiple Reaction Monitoring (MRM) transitions (application note 720005189EN, Waters). The instrument settings for nebulizer gas (Gas 1), turbogas (Gas 2), curtain gas, and collision gas were 40 psi, 60 psi, 30 psi, and medium, respectively. The interface heater was on, the Turbo V ESI source temperature was 600 °C, and the ionspray voltage was 5.5 kV. For all MRM transitions the values for declustering potential, entrance potential, and cell exit potential were 60 V, 10 V, and 10 V, respectively. The collision energies ranged from 10 to 50 V. The LC chromatogram peaks of internal standards and endogenous amino acid derivates were integrated using the Analyst 1.6.3 software (SCIEX). Endogenous amino acids were quantified on the basis of external calibration curves which were calculated from LC-MS/MS measurements of serially diluted synthetic amino acid standard solutions within the range of 0.0 to 27.5 pmol on column. To each dilution fixed amounts of the isotope-labeled internal standards were added. The amino acid calibration solutions were derivatized as described above for the tissue homogenates. The standard calibration curves were plotted based on molar concentration versus peak area ratio of amino acid standards to isotope-labeled internal standards. Linearity and correlation coefficients (R2) of the calibration curves were obtained via linear regression analysis. R2 of the calibration curves were >0.99. The calculated amounts of endogenous amino acids were normalized to the wet weight of the tissue sample.

**Electrophysiology**

*Animals and brain slice preparation*

Experiments were performed on brain slices from 25 - 33 days old male and female astro-DKO mice (n= 5) and L1^-/-^ mice (n= 3). The number of neurons analyzed in each experimental setting is indicated in the Fig. 6. Animals were lightly anesthetized with isoflurane (B506; AbbVie Deutschland GmbH and Co KG, Ludwigshafen, Germany) and subsequently decapitated. The brain was rapidly removed and the cerebellum was immediately dissected. Sagittal slices (300 μm) of the cerebellum were cut with a vibration microtome (HM-650 V; Thermo Scientific) under cold (4°C), carbogenated (95% O_2_ and 5% CO_2_), glycerol-based modified artificial cerebrospinal fluid (GaCSF). GaCSF contained (in mM): 250 Glycerol, 2.5 KCl, 2 MgCl_2_, 2 CaCl_2_, 1.2 NaH_2_PO_4_, 10 HEPES, 21 NaHCO_3_, 5 Glucose adjusted to pH 7.2 (with NaOH) resulting in an osmolarity of ~310 mOsm. Brain slices were transferred into carbogenated artificial cerebrospinal fluid (aCSF). First, they were kept for 20 min in a 35°C 'recovery bath' and then stored at room temperature (24°C) for at least 30 min prior to recording. aCSF contained (in mM): 125 NaCl, 2.5 KCl, 2 MgCl_2_, 2 CaCl_2_, 1.2 NaH_2_PO_4_, 21 NaHCO_3_, 10 HEPES, and 5 Glucose adjusted to pH 7.2 (with NaOH) resulting in an osmolarity of ~310 mOsm. Slices were transferred to a recording chamber (~3 ml volume) and continuously superfused with carbogenated aCSF at a flow rate of ~2 ml·min^-1^. Experiments were carried out at ~32°C using an inline solution heater (SH27B; Warner Instruments) operated by a temperature controller (TC-324B; Warner Instruments). Neurons in the cerebellum were visualized with a fixed-stage upright microscope (BX51WI; Olympus), using a 20x water immersion objective (XLUMplan FI; 20×; 0.95 numerical aperture; Olympus) with infrared-differential interference contrast (Dodt 1993). PCs were identified by the anatomical location in the cerebellum. Current-clamp recordings were performed with a modified ELC03-XS amplifier (NPI Electronic) controlled by the PatchMaster software (version 2.32; HEKA). Data were sampled at intervals of 20 μs (50 kHz) with a CED 1401 using Spike2 (both Cambridge Electronics). The liquid junction potential between intracellular and extracellular solution was compensated (14.6 mV; calculated with Patcher's Power Tools plug-in for Igor Pro 6 [Wavemetrics]).

*Perforated patch recordings*

Perforated patch recordings were performed using protocols modified from Horn & Marty (Horn and Marty 1988) and Akaike & Harata (Akaike and Harata 1994). Electrodes with tip resistances between 3 and 5 MOhm were fashioned from borosilicate glass (0.86 mm inner diameter; 1.5 mm outer diameter; GB150- 8P; Science Products) with a vertical pipette puller (PP-830; Narishige). Patch recordings were performed with ATP and GTP free pipette solution containing (in mM): 128 Kgluconate, 10 KCl, 10 HEPES, 0.1 EGTA, 2 MgCl_2_ and adjusted to pH 7.2 (with KOH). ATP and GTP were omitted from the intracellular solution to prevent uncontrolled permeabilization of the cell membrane (Lindau and Fernandez 1986). The patch pipette was tip filled with internal solution and back filled with 0.02% tetraethylrhodamine-dextran (D3308, Invitrogen) and amphotericin-containing internal solution (~200-250 μg·ml^-1^; G4888; Sigma) to achieve perforated patch recordings. Amphotericin was dissolved in dimethyl sulfoxide (final concentration: 0.1 - 0.3%; DMSO; D8418, Sigma) as described previously (Kyrozis and Reichling 1995); and was added to the modified pipette solution shortly before use. The used DMSO concentration had no obvious effect on the investigated neurons. During the perforation process access resistance (*R*a) was constantly monitored and experiments were started after *R*a and the action potential (AP) amplitude were stable (~15 – 20 min). A change to the whole-cell configuration was indicated by diffusion of tetraethylrhodamine-dextran into the neuron. Such experiments were rejected. To block GABAergic and glutamatergic synaptic input the aCSF contained 10^-4^ M picrotoxin (P1675; Sigma-Aldrich), 5 x 10^-5^ M D-AP5 (A5282; Sigma-Aldrich), and 10^-5^ M CNQX (C127; Sigma-Aldrich). The cell input resistance was calculated from voltage responses to small hyperpolarizing current pulses. To analyze excitability, i.e. evoked action potential firing, a series of depolarizing current pulses (100 pA to 1000 pA in 100 pA increments; 1.5 s duration) were injected. For each current pulse the number of action potentials was determined.

*Data analysis*

Data analysis was performed with Spike2 (Cambridge Electronics), Graphpad Prism (version 5.0b; Graphpad Software Inc) and Igor Pro.

*Single-cell labeling*

To label single cells, 1% biocytin (B4261, Sigma) was added to the pipette solution. After the recordings, the brain slices were fixed in Roti-Histofix (P0873, Carl Roth) for ∼12 h at 4°C and rinsed in 0.1 M PBS (pH 7.2, 3 times for 10 min each time). Brain slices were incubated in PBS containing 1% Triton X-100 and 10% normal goat serum (30 min, RT; Serva). Afterwards, the slices were incubated in Alexa Fluor 633 (Alexa 633)–conjugated streptavidin (1:400, 2h, RT; S21375, Molecular Probes) that was dissolved in PBS. Brains were rinsed in PBS (5 times for 10 min each time, RT), dehydrated, cleared, and mounted in Permount (SP-15, Thermo Fisher Scientific). Fluorescence images of brain slices were captured with a confocal microscope (SP-8, Leica) equipped with HCX-PL Fluotar × 10 (0.3 NA), HC PL APO ×20 (0.75 NA), and HC PL APO ×63 (1.2 NA) objectives. Streptavidin-Alexa 633 was excited with a He-Ne Laser at 633 nm, and emission was collected through a 650-nm LP filter. Scaling, contrast enhancement, and *z*-projections were performed with ImageJ v2.0.0. The final figures were prepared with Affinity Designer (Ver. 1.6.1, Serif Ltd).

**Supplementary References**

Akaike N, Harata N. 1994. Nystatin perforated patch recording and its applications to analyses of intracellular mechanisms. Jpn J Physiol 44:433-73.

Dodt HU. 1993. Infrared-interference videomicroscopy of living brain slices. Adv Exp Med Biol 333:245-9.

Horn R, Marty A. 1988. Muscarinic activation of ionic currents measured by a new whole-cell recording method. J Gen Physiol 92:145-59.

Kyrozis A, Reichling DB. 1995. Perforated-patch recording with gramicidin avoids artifactual changes in intracellular chloride concentration. J Neurosci Methods 57:27-35.

Lindau M, Fernandez JM. 1986. A patch-clamp study of histamine-secreting cells. J Gen Physiol 88:349-68.

**Supplementary Figures**

**
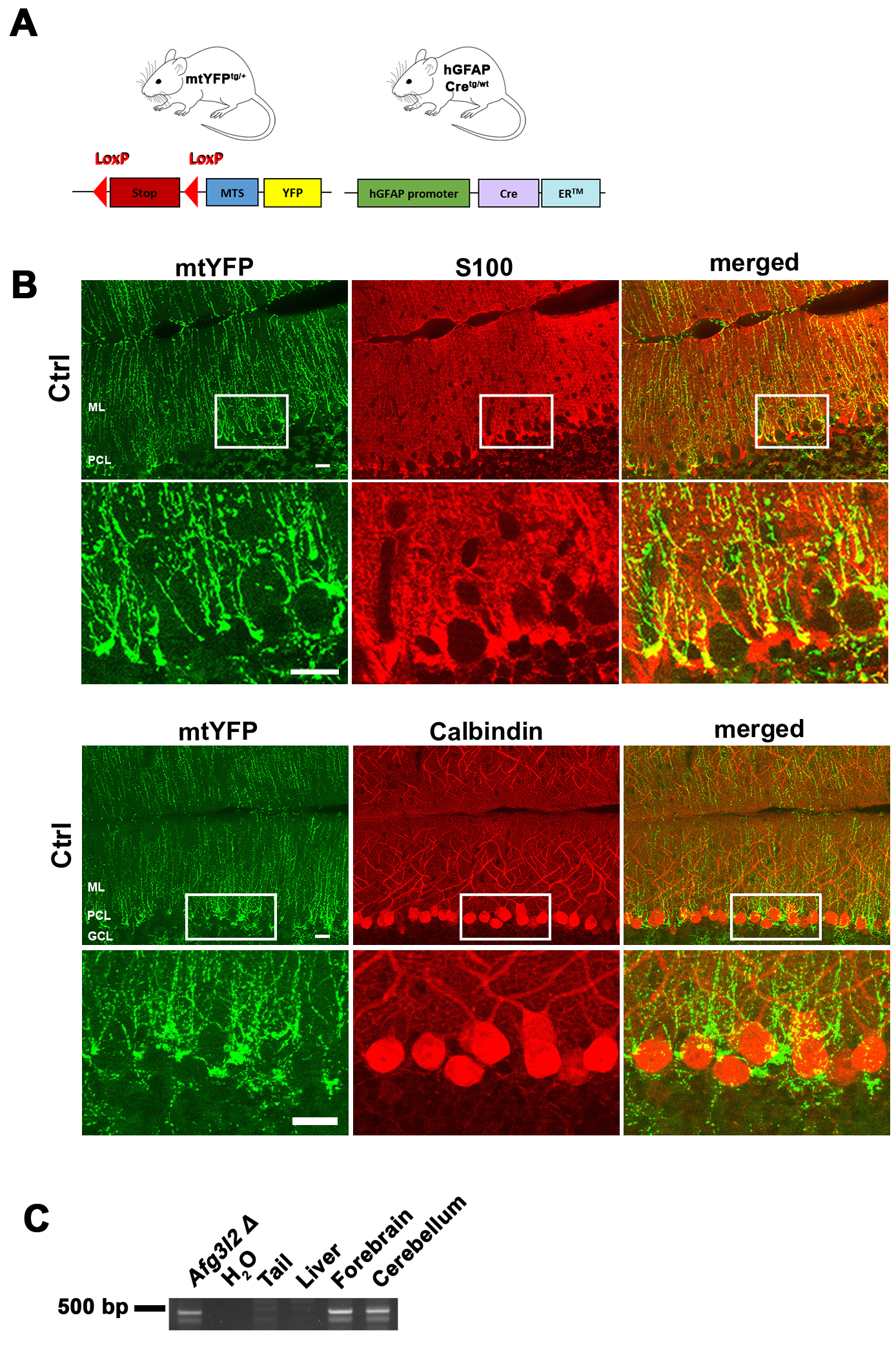
**

**Supplementary Figure 1. Specificity of recombination in BG in the cerebellum and efficiency of *Afg3l2* deletion**

(A) mtYFP reporter mice (mtYFP^tg/+^) were crossed with GFAP-Cre^tg/wt^ mice to reveal the efficiency of recombination after tamoxifen injection. (B) Immunostainings of S100 or calbindin and endogenous mtYFP immunofluorescence indicate that targeted cells are astrocytes and not PCs. Scale bars: 20 µm; (C) DNA extracted from tail, liver, forebrain and cerebellum of an astro-L2 KO male mouse at 8 weeks was analyzed by PCR to assess recombination. The band at 467 bp reveals the recombination occurring in the forebrain and cerebellum. A weak smeary band is observed in the tail.

**
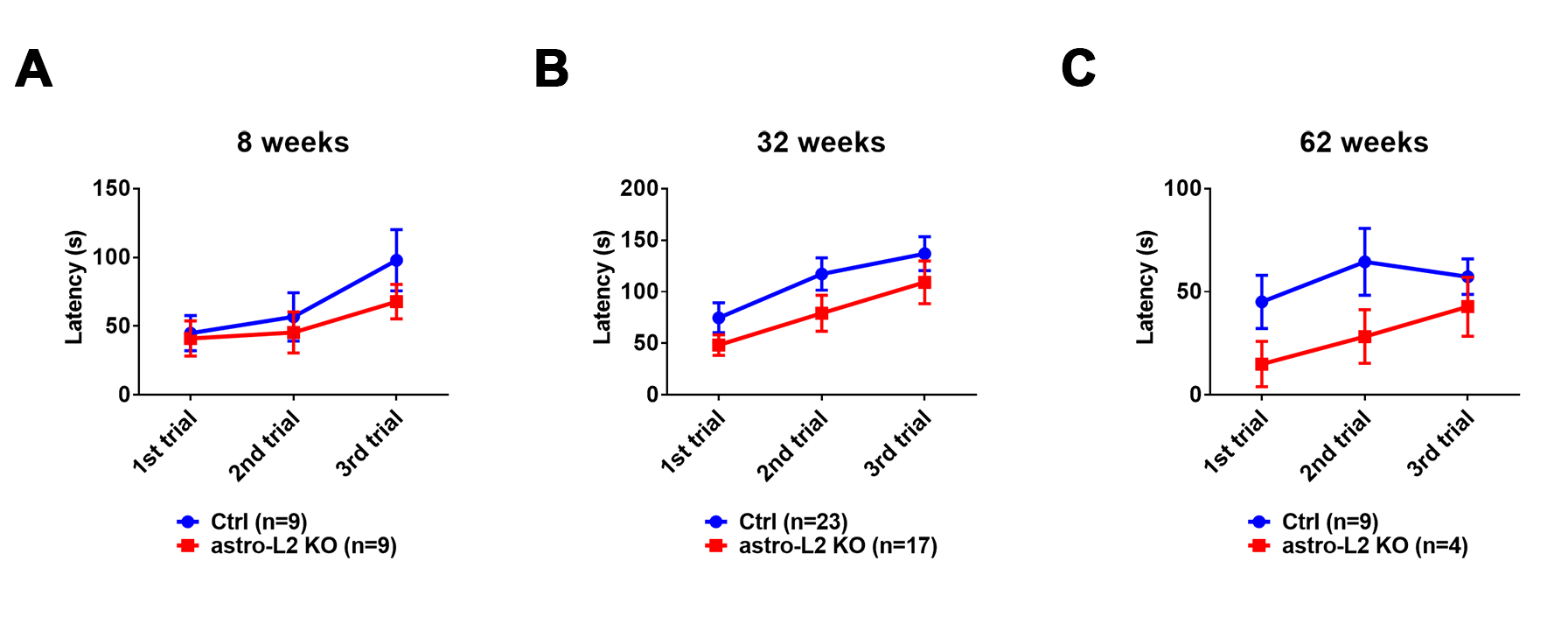
**

**Supplementary Figure 2. Rotarod test of female astro-L2 KO and Ctrl mice**

(A-C) Time on the rotarod apparatus before falling at different ages of astro-L2 KO and Ctrl female mice. Student’s t-test, error bars represent SEM.


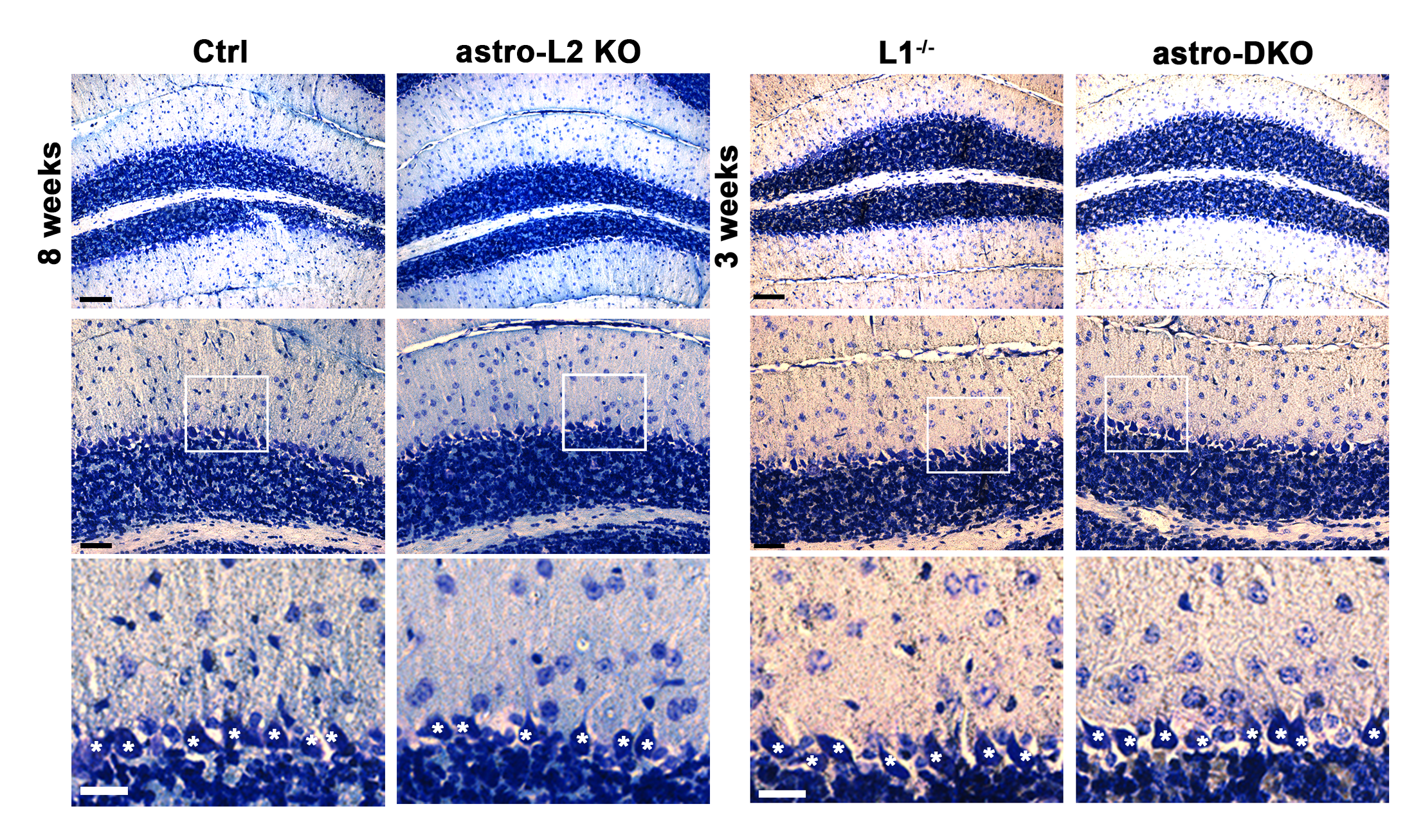


**Supplementary Figure 3. Gross histological characterization of the cerebellum**

Nissl stainings show normal development and layer organization of the cerebellum in both astro-L2 KO and astro-DKO mice. Scale bars upper panels: 100 µm; middle panels: 50 µm; lower panels: 25 µm. Asterisks mark PCs.


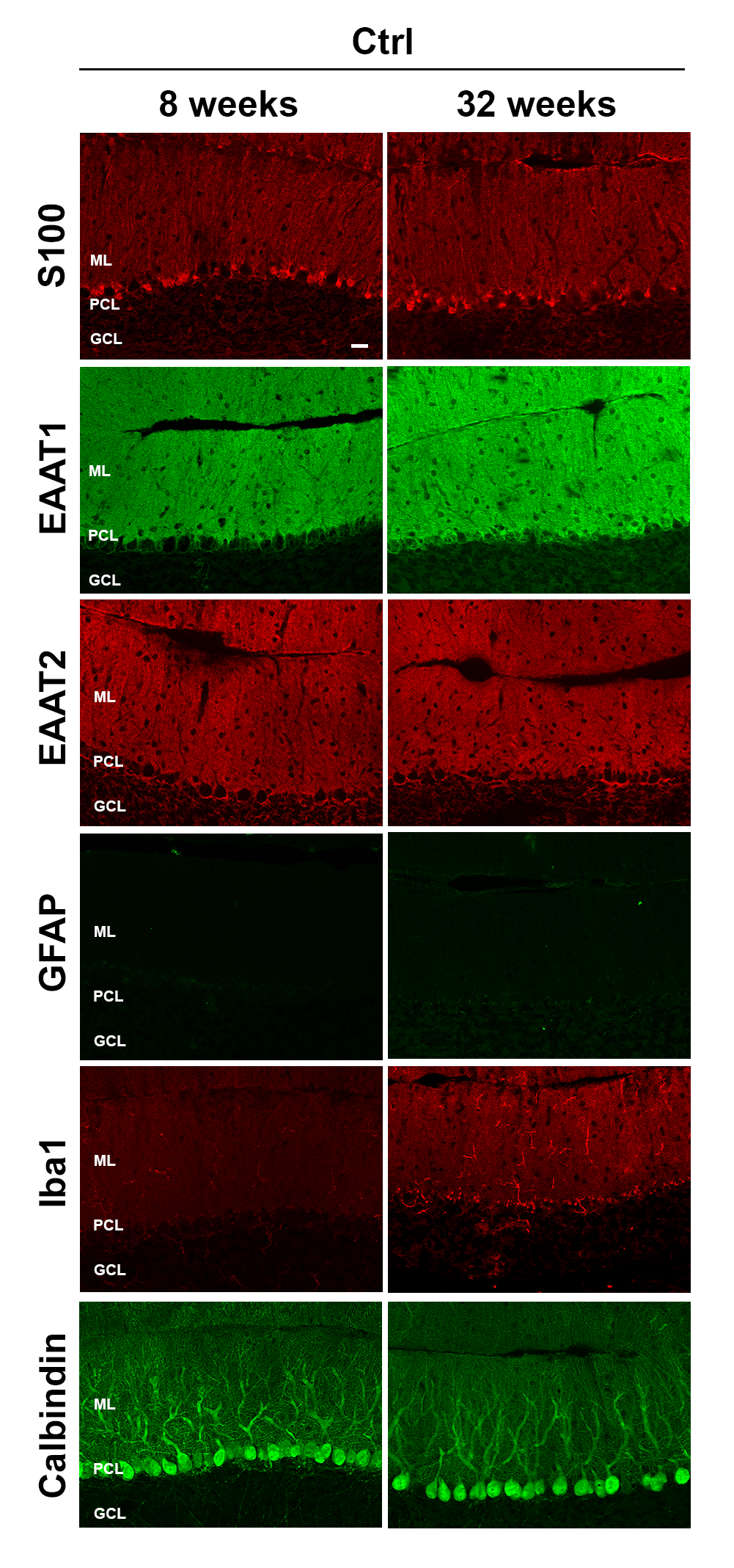


**Supplementary Figure 4. Control immunofluorescence in the cerebellum**

Immunostaining of Ctrl cerebellum at the indicated ages with different antibodies, as specified. No alterations were observed. Scale bar: 20 µm.


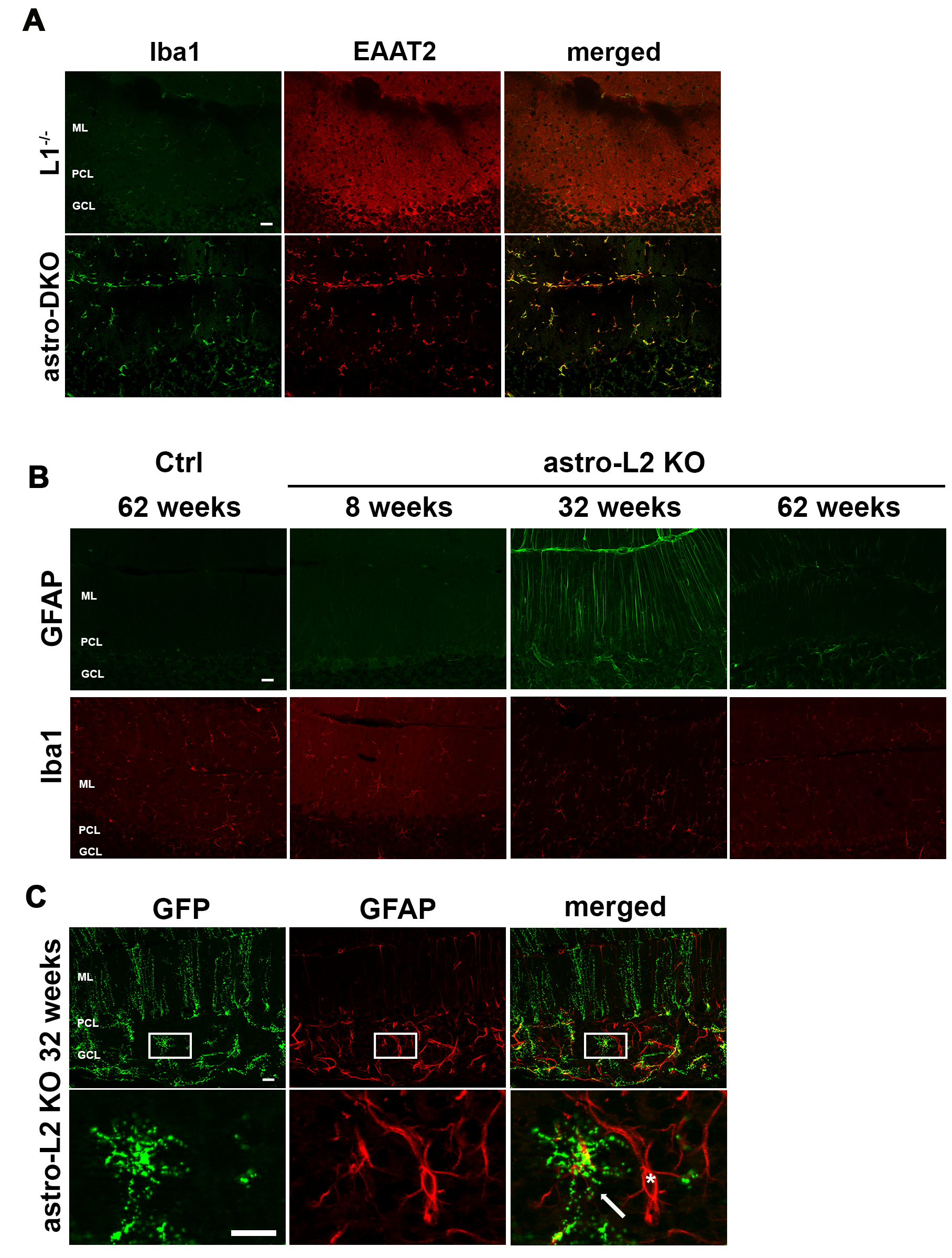


**Supplementary Figure 5. Inflammatory responses**

(A) Co-staining for Iba1 and EAAT2 shows that EAAT2 is expressed mainly in microglial cells in astro-DKO mice at 4 weeks (n=3/group). Scale bar: 20 µm. (B) Immunostaining for GFAP and Iba1 at different time-points in astro-L2 KO (n=3 for each group at each time-point). (C) Co-staining of GFAP and mtYFP in 32 weeks old astro-L2 KO (n=3 at each time-point for each group). Most reactive astrocytes are positive for mtYFP (arrow). Asterisk indicates a GFAP reactive astrocyte with no mtYFP signal. Scale bar: 20 µm; scale bar enlargements: 20 µm.

**
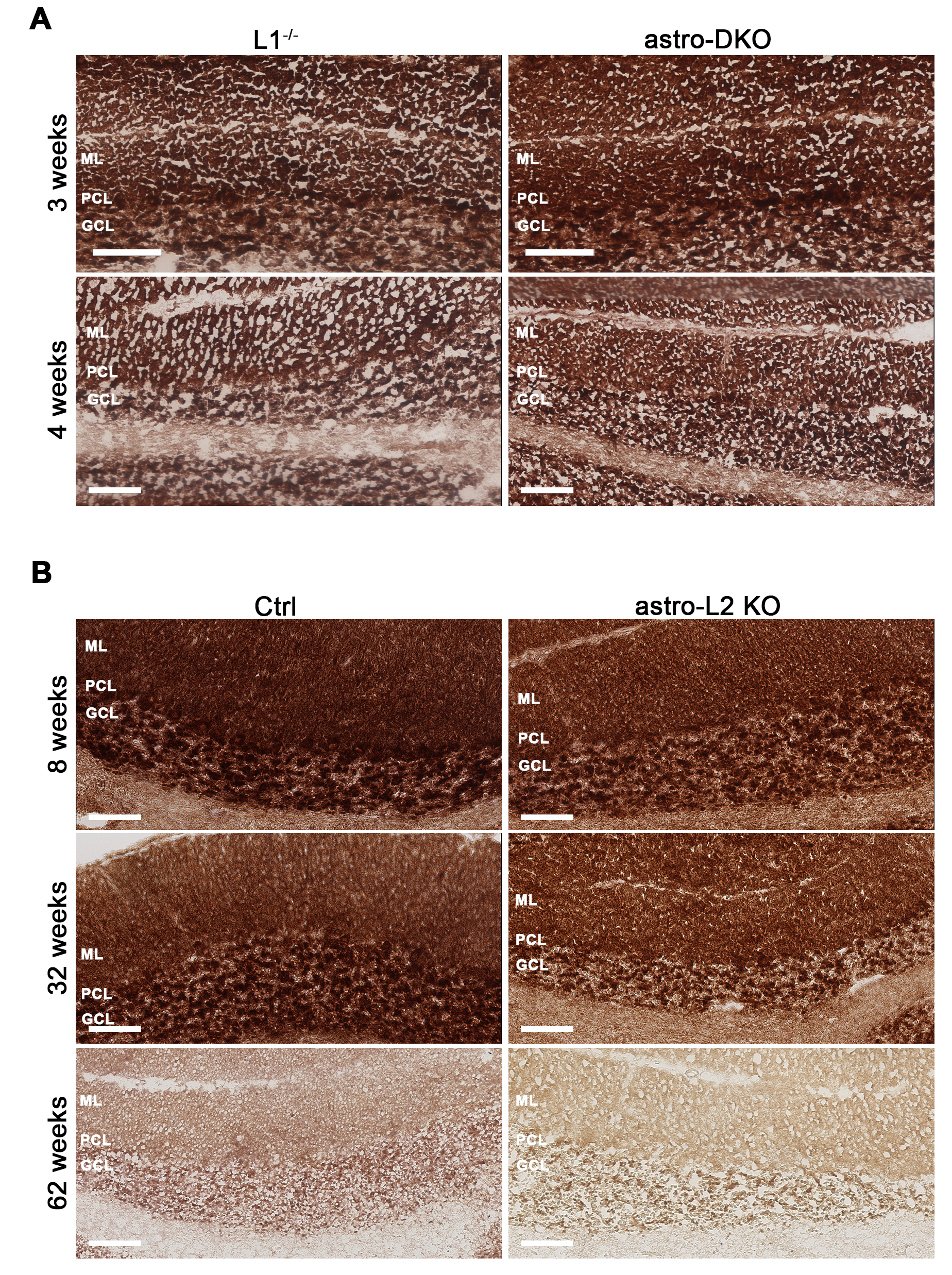
**

**Supplementary Figure 6. *m*-AAA protease deletion in astrocytes does not cause COX deficiency**

(A-B) COX-SDH staining of cerebellar slices revealed no sign of respiratory deficiency in either astro-L2 KO or astro-DKO mice (n≥3 for each group at each time-point). Scale bars: 100 µm.

**
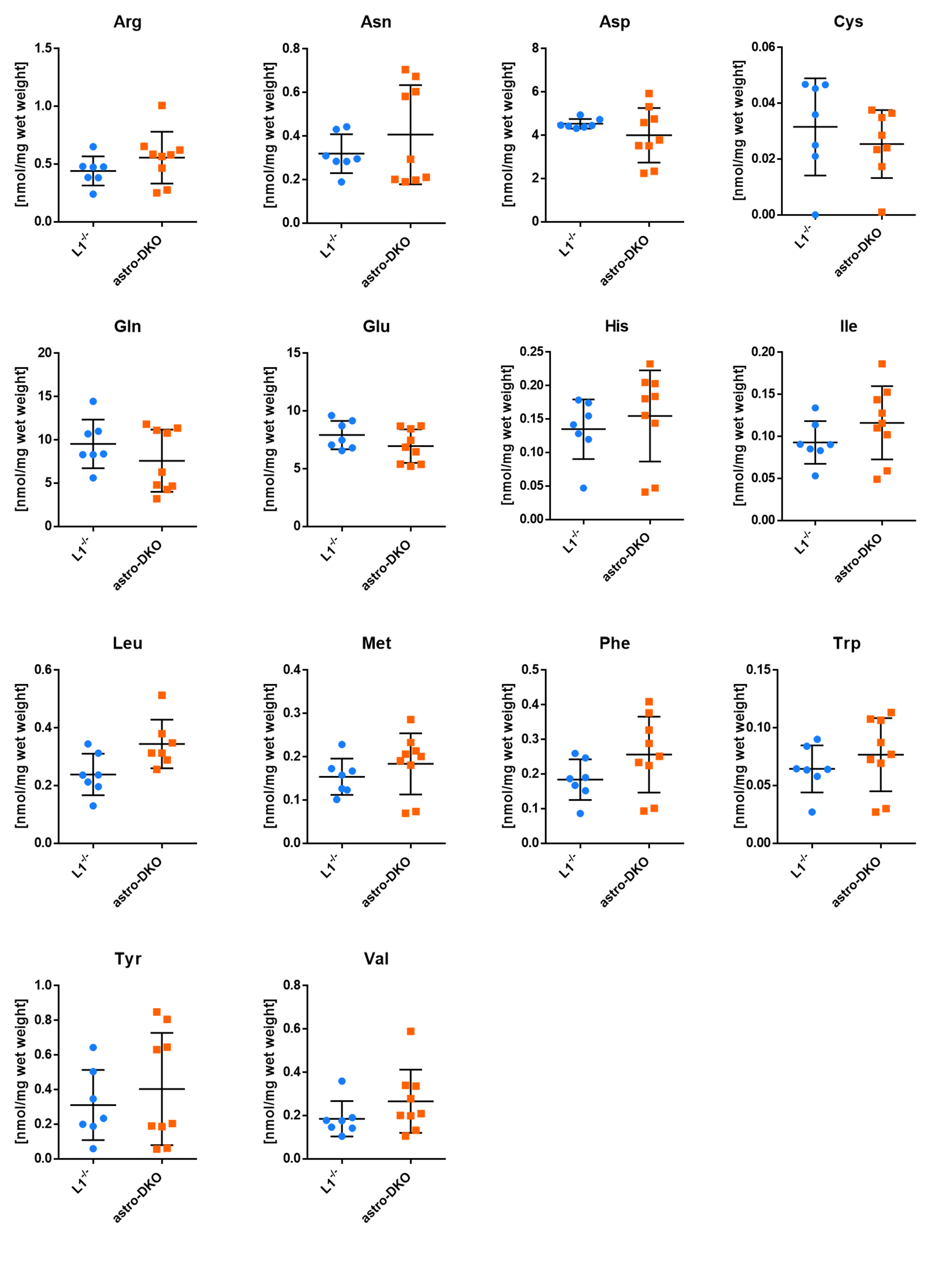
**

**Supplementary Figure 7. Additional amino acid quantification**

Amino acid levels in cerebellar lysates from astro-DKO and L1^-/-^ mice at 4 weeks, visualized as dot plots, including mean ± SD.

**
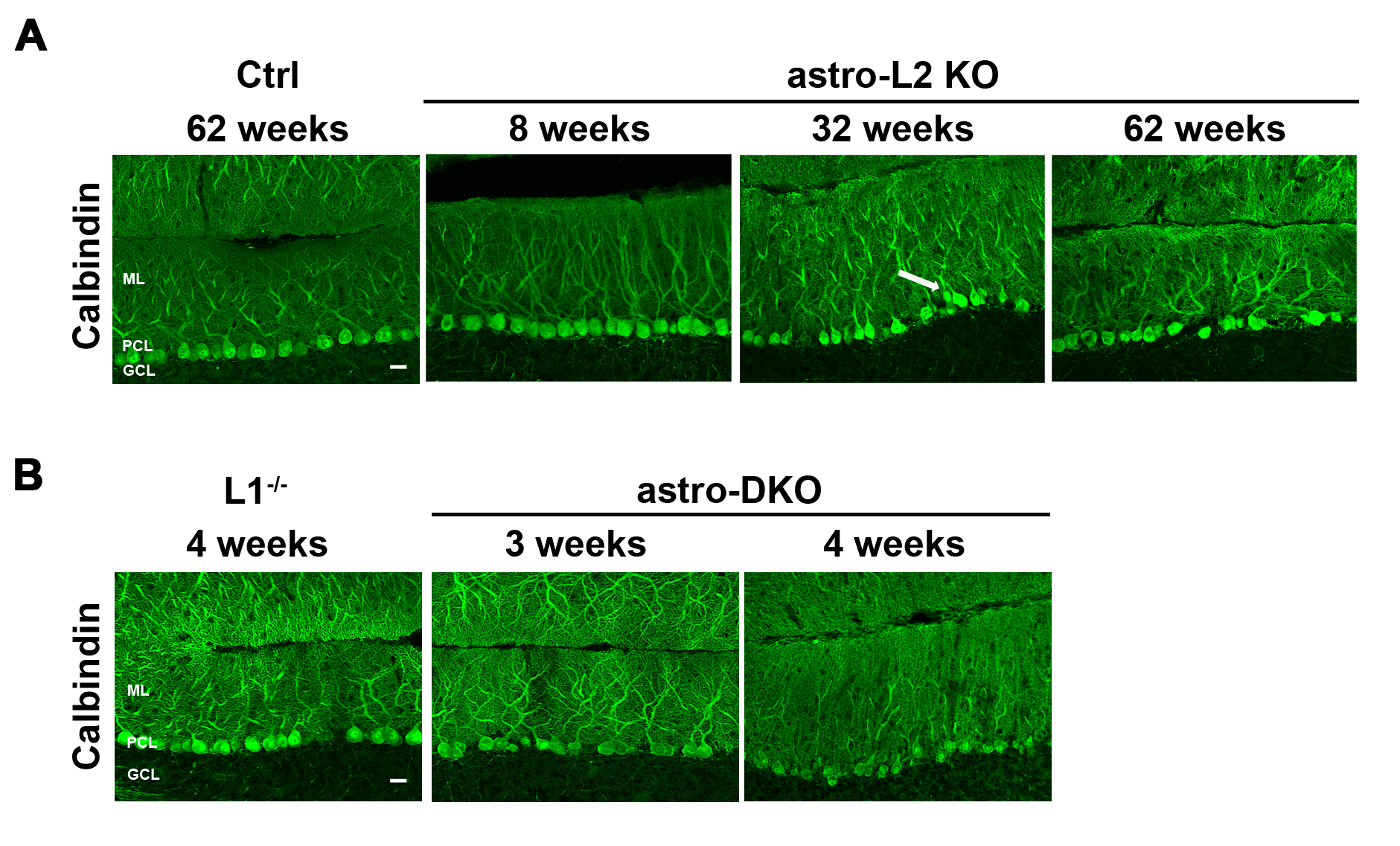
**

**Supplementary Figure 8. Secondary morphological changes in PCs.**

(A-B) Immunostaining for calbindin in astro-L2 KO (A) and astro-DKO (B) at different time-points (n at least 3 for each group at each time-point). Arrow indicates a PC cell body moving in the ML. Scale bar: 20 µm.
