# Supplementary material for: Astrocyte‐specific deletion of the mitochondrial m‐AAA protease reveals glial contribution to neurodegeneration
Source: Glia. 2019 Apr 16;67(8):1526–41. doi: 10.1002/glia.23626 (PMC6618114; doi:10.1002/glia.23626)
Supplement: Supplementary file 2 — Appendix S2: Supplementary material for the Reviewers [file GLIA-67-1526-s002.docx]

**Supplementary material for the Reviewers**


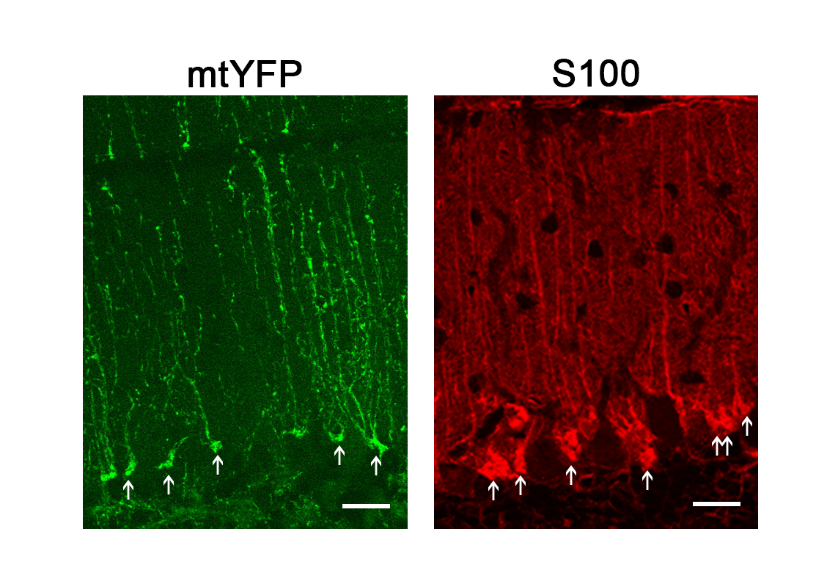

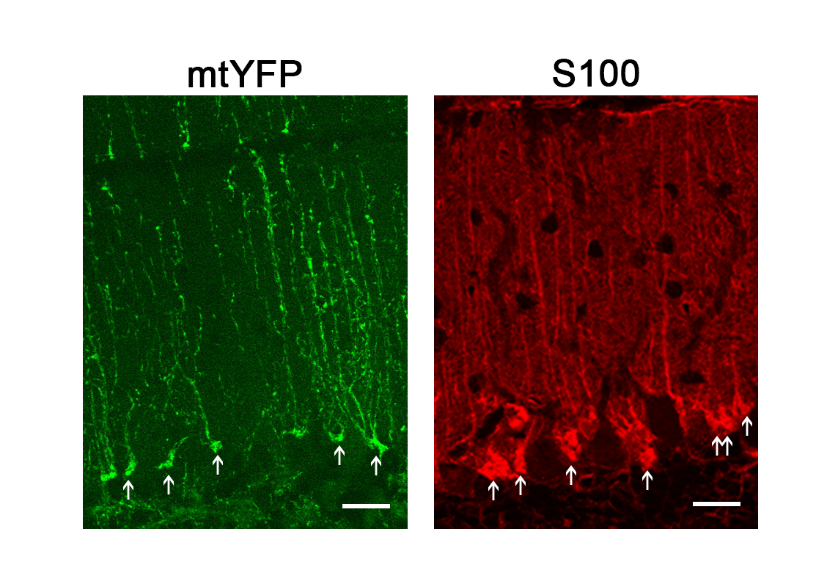


Examples of enlarged images of S100- and mtYFP-labelled BG used for quantification. Arrows show cell bodies.
